# Does training with blurred images bring convolutional neural networks closer to humans with respect to robust object recognition and internal representations?

**DOI:** 10.3389/fpsyg.2023.1047694

**Published:** 2023-02-15

**Authors:** Sou Yoshihara, Taiki Fukiage, Shin'ya Nishida

**Affiliations:** ^1^Department of Intelligence Science and Technology, Graduate School of Informatics, Kyoto University, Kyoto, Japan; ^2^NTT Communication Science Laboratories, Nippon Telegraph and Telephone Corporation, Atsugi, Japan

**Keywords:** convolutional neural networks, object recognition, visual development, perceptual organization, optical blur

## Abstract

It has been suggested that perceiving blurry images in addition to sharp images contributes to the development of robust human visual processing. To computationally investigate the effect of exposure to blurry images, we trained convolutional neural networks (CNNs) on ImageNet object recognition with a variety of combinations of sharp and blurred images. In agreement with recent reports, mixed training on blurred and sharp images (B+S training) brings CNNs closer to humans with respect to robust object recognition against a change in image blur. B+S training also slightly reduces the texture bias of CNNs in recognition of shape-texture cue conflict images, but the effect is not strong enough to achieve human-level shape bias. Other tests also suggest that B+S training cannot produce robust human-like object recognition based on global configuration features. Using representational similarity analysis and zero-shot transfer learning, we also show that B+S-Net does not facilitate blur-robust object recognition through separate specialized sub-networks, one network for sharp images and another for blurry images, but through a single network analyzing image features common across sharp and blurry images. However, blur training alone does not automatically create a mechanism like the human brain in which sub-band information is integrated into a common representation. Our analysis suggests that experience with blurred images may help the human brain recognize objects in blurred images, but that alone does not lead to robust, human-like object recognition.

## 1. Introduction

Human visual acuity, evaluated in terms of the minimum angle of resolution or the highest discernable spatial frequency, is affected by a variety of processes including eye optics, retinal sensor sampling, and the subsequent neural signal processing. In daily visual experiences, visual acuity changes depending on, for example, the degree to which the current focal length of the eye agrees with the distance to the target object, or whether the target is sensed at the fovea, where image sampling is dense, or at far-peripheral vision, where sparse image sampling is followed by spatial pooling. Visual acuity also changes progressively with each stage of development. Infants who are born with low visual acuity gradually acquire near adult-level acuity within the first few years of life (Dobson and Teller, 1978; Banks and Salapatek, 1981). Considering that the loss of visual acuity can be approximated by blurring the image by low-pass (high-cut) filtering, one can say that most humans have a rich experience seeing blurred visual images in addition to sharp ones.

It has been suggested that the experience of blurred visual images might be functionally beneficial, enabling the visual system to use global configural structures in image recognition (Grand et al., 2001; Le Grand et al., 2004; Vogelsang et al., 2018). Several recent studies test this hypothesis computationally by machine learning using artificial neural networks (Vogelsang et al., 2018; Katzhendler and Weinshall, 2019; Avberšek et al., 2021; Geirhos et al., 2021; Jang and Tong, 2021, 2022). Vogelsang et al. (2018) trained a convolutional network (CNN) to recognize human faces. To simulate how visual acuity gradually improves during the initial stage of life, they changed the training images from blurred to sharp ones during training (B2S) and found that the network achieves robust face recognition for a wide range of image blur, as humans do. In contrast, the network can only recognize sharp images when trained on sharp images. The network can only recognize blurred images when trained using blurred images or sequentially trained on images that change from sharp to blurred. Jang and Tong (2021) found that the effect of B2S training is task-specific. It leads to blur-robust recognition for face recognition as Vogelsang et al. reported, but not for object recognition. Avberšek et al. (2021) was also unable to obtain blur-robust object recognition by means of B2S training. However, object recognition achieves blur robustness when blurred and sharp images are always mixed during training (B+S).

With a similar research motivation in mind, we examined the effects of blur training on object recognition by CNNs. We investigated which types of blur training make the CNNs sensitive to coarse-scale global features as well as fine-scale local features, and bring them closer to the human object recognition system. We evaluated the object-recognition performance of the blur-trained CNNs not only using low-pass filtered test images, but also for other types of images including band-pass filtered images and shape-texture cue conflict images (Geirhos et al., 2019) to ascertain whether blur training affects global configurational processing in general. In agreement with previous reports (Avberšek et al., 2021; Jang and Tong, 2021), our results show that B+S training, but not B2S training, leads to blur-robust object recognition comparable to human performance. However, B+S training is not sufficient to produce robust human-like object recognition based on global configuration features. For example, it reduces the texture bias of CNNs for shape-texture cue conflict images, but the effect is too small to achieve a strong shape bias comparable to that of humans.

In the latter half of this report, using correlation analyses of internal representations and zero-shot transfer learning, we examine how B+S training makes CNN less affected by image blur. Our results suggest that initial low-pass filtering contributes to the blur robustness of B+S-Net, but only partially. Representational similarities in the intermediate layers suggest that B+S-Net processes sharp and blurry images not through separate specialized sub-networks, but through a common blur-robust mechanism. Furthermore, we found that B+S training for other object labels transfers to another label trained only with blurred or sharp images, which suggests that B+S training lets the network learn general blur-robust features. However, blur training alone does not automatically create a mechanism like the human brain where sub-band information is integrated into a common representation.

Overall, our results suggest that experience with blurred images may help the human brain develop neural networks that recognize the surrounding objects regardless of image blurring, but that alone does not lead to robust, human-like object recognition.

## 2. Methods

We investigated the performance of several training methods with a mixture of blurred images. In the experiments, we mainly used 16-class-ImageNet (Geirhos et al., 2018) as a dataset, and the analysis is based on 16-class-AlexNet, with 16 final layer units. However, we also ran some of the experiments using a 1000-class-ImageNet and tested other network architectures to ensure the generalizability of our results. A list of the networks compared in this study is summarized in Table 1. We trained all the models from scratch except for SIN-trained-Net (Geirhos et al., 2019), for which we used the pre-trained model provided by the authors. We did not fine-tune any of the models for the test tasks. Further, we collected human behavior data via Amazon Mechanical Turk (AMT) to compare human performances with those of our blur-trained models. Below, we provide in detail information about the dataset, model architecture, training strategies, and a human behavior study.

**Table 1:** CNN models used in this study.

**Model name**	**Architecture**	**Number of units in final layer**	**Training dataset**	**Pre-training**	**Fine-tuning**
16-class-AlexNet	AlexNet	16	16-class-ImageNet (Geirhos et al., 2018)	No	No
1000-class-AlexNet	AlexNet	1,000	ImageNet(ILSVRC2012)	No	No
VOneNet	VOneBlock (Dapello et al., 2020) + AlexNet	16	16-class-ImageNet (Geirhos et al., 2018)	No	No
16-class-VGG16	VGG16 (Simonyan and Zisserman, 2015)	16	16-class-ImageNet (Geirhos et al., 2018)	No	No
16-class-ResNet50	ResNet50 (He et al., 2016)	16	16-class-ImageNet (Geirhos et al., 2018)	No	No
SIN-trained-Net (Geirhos et al., 2019)	AlexNet	1,000	SIN-ImageNet (Geirhos et al., 2019)	Yes	No

### 2.1. Dataset: 16-class-ImageNet

In order to facilitate comparison with experimental data on humans, we used the 16-class-ImageNet dataset. This dataset was created by Geirhos et al. (2018), who grouped 1,000 ImageNet classes into superior classes such as “dog" and “clock" and selected the following 16 classes from them: *airplane, bear, bicycle, bird, boat, bottle, car, cat, chair, clock, dog, elephant, keyboard, knife, oven*, and *truck*. There are 40,517 training images and 1,600 test images. There was no overlap between them. The image size is 224 × 224 × 3 (height, width, color). The performance of the model trained on the regular 1000-class-ImageNet is also investigated in a later section (section 3.3.3).

### 2.2. CNN model: 16-class-AlexNet

We chose AlexNet (Krizhevsky et al., 2012) as the CNN model for our main analysis. We used the model architecture provided in a popular deep learning framework, Pytorch, and trained the model from scratch. To match the number of classes in the 16-class-ImageNet, we changed the output number in the final layer from 1000 to 16.

We chose AlexNet because of its similarity to the hierarchical information processing of the human visual cortex. For example, the visualization of filters in the first layer of AlexNet trained with ImageNet shows the formation of various Gabor-like filters with different orientations and scales (Krizhevsky et al., 2012). The Gabor functions are known to be good approximations of the spatial properties of V1 simple cell receptive fields (Jones and Palmer, 1987). In section 3.3.4, we also analyze a model that explicitly incorporated the Gabor filters as the initial layer of AlexNet using VOneBlock proposed by Dapello et al. (2020). A study of the brain hierarchy (BH) score, which takes into account the hierarchical similarity between the deep neural network (DNN) and the brain, shows that AlexNet has a high BH score (Nonaka et al., 2021). The information representation in the convolutional layer of AlexNet corresponds to the lower visual cortex of the brain, while the fully connected layer corresponds to the higher visual cortex of the brain. In addition, AlexNet is an easy model to interpret in that it has a small number of layers and does not contain complex operations such as Skip Connection.

### 2.3. Training with blurred images: Blur training

**Figure 1:**
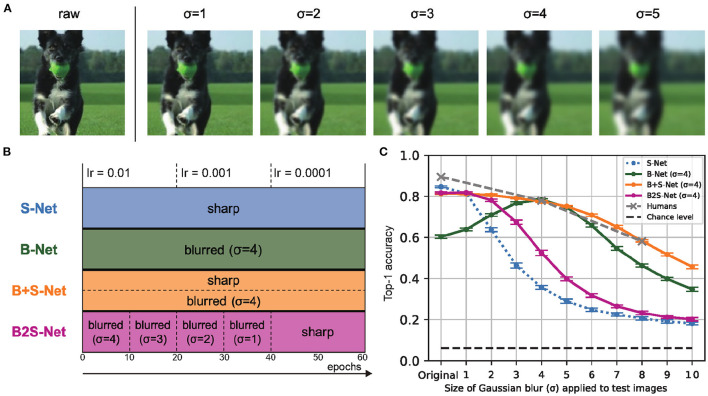
Blur training brings CNN closer to humans in recognizing low-pass filtered object images. **(A)** Sample blurred images. **(B)** Four blur training methods. **(C)** Classification accuracy on low-pass filtered test images. Top-1 accuracy is the rate at which the model's first choice matches the expected answer. Error bars represent 95% confidence intervals estimated from the performances of eight models trained with different random seeds.

In this experiment, in addition to the regular training, we trained CNNs with blurred images using three different strategies (Figure 1). We used Gaussian kernel convolution to blur images. The blur size was manipulated by changing the standard deviation (σ) of the Gaussian kernel as shown in Figure 1A. The spatial extent of the Gaussian kernel (*k*) was determined depending on σ as follows: *k* = Round(8σ+1).^1^ When *k* was an even number, one was added to make it an odd number.

In the following, we refer to the trained models as S-Net, B-Net, B+S-Net, and B2S-Net, respectively, depending on which image blurring strategy was used in training (Figure 1B). Unless otherwise stated, the architecture of each model is 16-class-AlexNet. We trained all the models for 60 epochs (the number of training cycles through the full training dataset), with a batch size of 64. The optimizer was stochastic gradient descent (SGD) with momentum = 0.9 and weight decay = 0.0005. The initial learning rate (lr) was set to 0.01 and decreased by a factor of ten at every 20 epochs. The number of training images was 40,517, the same for all models, and we applied random cropping and random horizontal flipping to all training images. The image size was 224 × 224 × 3 (height, width, color). We used PyTorch (version 1.2.0) and one of two GPU machines to train each model. The GPU environments were Quadro RTX 8000 (CUDA Version: 10.2) and GeForce RTX 2080 (CUDA Version: 10.2).

- **S-Net** is a model trained on sharp (original, unblurred) images.
- **B-Net** In the training of B-Net, all the training images were blurred throughout the entire training period. We mainly discuss the performance of the model trained with a fixed blur size of σ = 4 px.
- **B+S-Net** In the training of B+S-Net, we blurred half of the samples randomly picked in each batch of training images throughout the entire training period. We mainly discuss the performance of the model trained with a fixed blur size of σ = 4 px. The performance of B+S-Net trained with randomly varied σ is presented in section 3.3.2.
- **B2S-Net** In the training of B2S-Net, the training images were progressively made sharper from a strongly blurred to the original, non-blurred image. Specifically, we started with a Gaussian kernel of σ = 4 px and decreased σ by one every ten epochs so that only sharp images without any blur were fed into the model in the last 20 epochs. This training method is intended to simulate human visual development and to confirm the effectiveness of starting training with blurred images, as claimed by Vogelsang et al. (2018).

To ensure the reproducibility of the results, we trained each network models with eight different initial weights, and computed the mean and the 95% confidence intervals for each condition.

### 2.4. Human image classification task

We collected human data using Amazon Mechanical Turk (AMT). We asked participants to perform an image classification task to investigate the difference between the models trained in this study and human image recognition capabilities.

**Figure 2:**
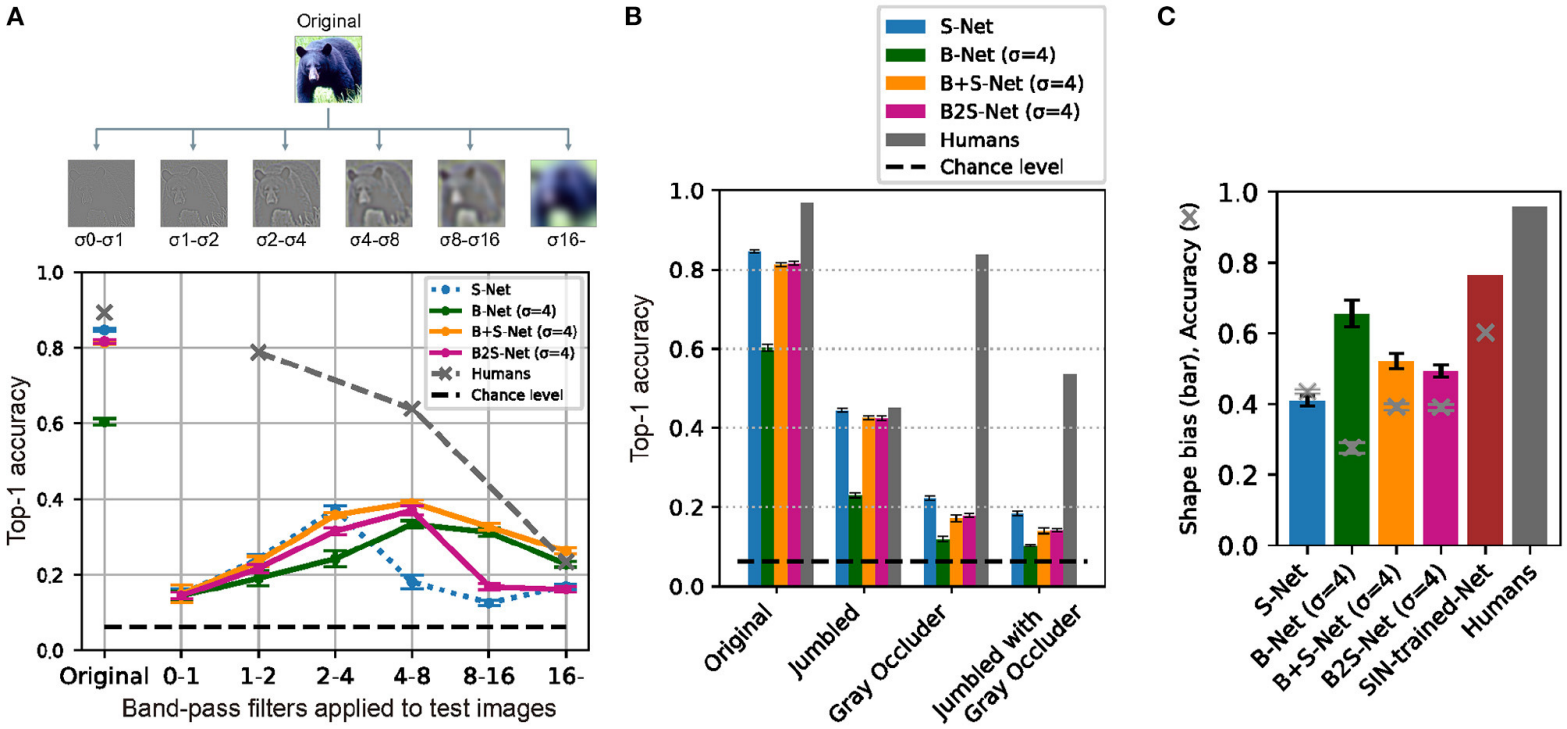
Blur training does not bridge the gaps between CNN and humans in recognition of **(A)** band-pass filtered object images, **(B)** jumbled/occluded images (Keshvari et al., 2021), and **(C)** shape-texture-cue-conflict images (Geirhos et al., 2019). The ordinate is the Top-1 accuracy of object classification. The exception is **(C)**, where shape bias is shown by bars, and classification accuracy on Stylized ImageNet (SIN) by crosses. Human accuracy for **(B, C)** are from the original studies. Error bars represent 95% confidence intervals estimated from the performances of eight models trained with different random seeds.

As stimuli for the classification task, we used the same 16-class-ImageNet test set that we used for evaluating CNN models (1,600 images, 100 images per class). In addition to the original test images, we tested the low-pass and band-pass versions of the 16-class-ImageNet test images for stimuli. The low-pass images were created by applying Gaussian kernel convolution while manipulating the standard deviation of the Gaussian kernel (σ) in the same manner as when we blurred the training images for CNN models. The band-pass images were created by taking the difference between two low-pass images obtained by blurring the same image with different σ (Figure 2A). In total, there were six conditions as follows: original image, low-pass image σ = 4 px, low-pass image σ = 8 px, low-pass image σ = 16 px, band-pass image σ1−σ2 (i.e., band-pass image obtained by subtracting the lowpass image with σ = 2 px from that with σ = 1 px), and band-pass image σ4−σ8.

For each task, one of the stimuli was presented and participants chose the category of an object in the image from 16 options. We had 170 subjects solve the tasks and obtained 6,817 pieces of categorization data (Original image: 1,108, low-pass image σ = 4: 1,103, low-pass image σ = 8: 1,124, low-pass image σ = 16: 1,134, band-pass image σ1−σ2: 1,188, band-pass image σ4−σ8: 1,160). Participants could complete the task for an arbitrary number of images. The consent form for the experiment was created using a Google Form, and a link to it was placed on the AMT task page. Each participant was asked to read the linked consent form and fill in the required information to register his or her consent. Experimental procedures were approved by the Research Ethics Committee at Graduate School of Informatics, Kyoto University, and were conducted in accordance with the Declaration of Helsinki.

## 3. Blur training: Results

We measure the model's performance on various test images and compare it to human performance to investigate what visual functions are acquired via blur training. First, we examined the classification accuracy for low spatial frequency images and analyzed whether the models could recognize coarse-scale information. Then, we examined whether the robustness to blurry images acquired through blur training could generalize to other types of robustness measured by using band-pass filtered images, images with manipulated spatial configurations of local elements, and shape-texture cue conflict images.

### 3.1. Recognition performance for low-pass images

In this section, we compare the image recognition performance for low spatial frequency images. Since the low-frequency information can capture global image features to some extent, the results of this task are expected to indicate, at least partially, whether the model recognizes global information or not. For this purpose, we examined the percentage of correct classifications for each model when the test image was blurred at different intensities. The test images are the test set of the 16-class-ImageNet containing 1,600 images. We also collected human classification task data using the same test images. The details are described in section 2.4.

The results of the above experiments are shown in Figure 1C. First, S-Net trained only on standard clear images shows a sharp drop in the accuracy when the image is strongly blurred. The B-Net's performance is high only for the blur level used in training (σ = 4) and blurs of similar strength. B2S-Net did not show much improvement in blur tolerance.

On the other hand, in the low-frequency image recognition test, B+S-Net, which was trained on both blurred and sharp images simultaneously, was able to recognize a wide range of features from sharp images to blurred images of various intensities (blur robustness). The robustness of B+S-Net against blur is similar to that indicated by human behavior data.

B2S-Net showed only a tiny improvement in terms of accuracy over S-Net, and B+S-Net showed stronger blur robustness than B2S-Net.

### 3.2. Recognition performance for other types of image manipulations

In the previous section, we showed that the models trained on both low-pass-filtered and sharp images acquire robustness to a broad range of image blur strengths. To gain a more detailed insight into what visual functions were acquired by blur training, we investigated the behavior of blur-trained models for image manipulations that were not used in the training procedure.

#### 3.2.1. Recognition performance for band-pass images

First, we used band-pass images to investigate the recognition performance of the model in each frequency band. The band-pass images were created by subtracting the two low-pass images of different σ. Using these band-pass images, we were able to find out in which frequency band blur training is influential. We also analyzed whether there is a difference between CNNs and humans regarding the frequency bands they can use for object recognition.

In our experiments, we performed a classification task on band-pass images for CNN models and humans, respectively. In the human experiments, we used AMT to conduct the image classification task for band-pass images σ1−σ2 and σ4−σ8. The details are described in section 2.4.

The results (Figure 2A) show that B+S-Net improves the accuracy of image recognition over a broader frequency range than the other models, and this indicates that training on blurred images is effective in acquiring the ability to recognize a broader range of frequency features. However, it did not show much effect on images in the high-frequency band.

Next, we compared the accuracy of humans and CNNs. The CNN model showed a lower recognition rate for band-pass stimuli, especially in the high-frequency range. Blur training does not lead to robust, human-like object recognition for bandpass images.

#### 3.2.2. Recognition performance for global configuration made of local patches

We further investigated whether blur training could change the global information processing in CNN models by using the test procedure proposed by Keshvari et al. (2021).

Keshvari et al. (2021) tested the difference in recognition performance between humans and CNNs by manipulating local patches. They divided the original image into several square tiles. There were four partitioning scales: (4 × 4), (8 × 8), (16 × 16), (32 × 32). A *Jumbled* image was one in which tiles were randomly replaced horizontally, preserving local information but distorting global shape and configural relationships. The *Gray Occluder* image, in which tiles were alternately grayed out, preserved the global shape and configural information but lost some local information. The *Jumbled with Gray Occluder* image combined the operations of the *Jumbled* and *Gray Occluder* images, and both local and global information were destroyed.

Keshvari et al. (2021) compared the difference in recognition accuracy between a CNN (VGG16 pre-trained on ImageNet without blur traning) and human observers using 640 images from eight classes in ImageNet. They found the pretrained CNN showed a significant decrease in accuracy for the *Jumbled* image, and the larger decreases for the *Gray Occluder* image, and the *Jumbled with Gray Occluder* image. Humans also showed a similar magnitude of decrease in accuracy for the *Jumbled* image, but only a small decrease for the *Gray Occluder* image. Their findings suggest that humnans can, but the pretrained CNN cannot, make use of global configural information preserved in the *Gray Occluder* image for object recognition.

In this study, we generated the jumbled/occluded images from the test images of the 16-class ImageNet in the same way as in Keshvari et al. (2021) and investigated whether the recognition performance of CNNs becomes closer to that of humans by blur training (Figure 2B). The results showed that training on blurred images did not change the overall trend of recognition performance on this test set. B+S-Net and B2S-Net did not improve the accuracy for *Gray Occluder* images compared to S-Net. These results suggest that the CNN models failed to utilize the global configural information preserved in the *Gray Occluder* image even after blur training.

#### 3.2.3. Recognition performance for texture-shape cue conflict images

To investigate whether the blur-trained models show a preference for shape information or texture information, we tested the shape bias proposed by the work of Geirhos et al. (2019).

Geirhos et al. (2019) created a texture-shape cue conflict image dataset where the texture information of one image was replaced by that of another image in a different class by using the style transfer technique of Gatys et al. (2016).^2^ The dataset consists of the same 16 classes as in the 16-class-ImageNet while each image has two correct labels based on its match to the shape or texture class. In total, the dataset contains 1,200 images (75 images per class). The shape bias measures how often the model answers the shape class when it correctly classifies a cue conflict image into either the shape or texture class, and is calculated by the following equation:


$$shape\;bias=\frac{correct\;shape\;decisions}{correct\;shape\;decisions+correct\;texture\;decisions.}$$

According to the results of Geirhos et al. (2019), while humans showed strong shape bias, CNN models trained on ImageNet showed weak shape bias (in other words, they showed texture bias). When the CNN models were trained on the Stylized-ImageNet (SIN) dataset, in which the texture information of an image was made irrelevant to the correct label by replacing the original texture with that of a randomly selected painting, the shape bias of the CNN models (SIN-trained-Net) became closer to that of humans. Moreover, we found SIN-trained-Net has a higher recognition rate for high-pass and band-pass images as humans do (Supplementary Figure S2B). However, training with SIN is biologically implausible and therefore not helpful in modeling the development of the human visual system.

Here, we calculated the shape bias of the models trained in our study using the texture-shape cue conflict image dataset provided by the authors of Geirhos et al. (2019) to see whether the blur training could enhance the shape bias of CNNs. Figure 2C presents the shape bias of the four models we trained as well as those of SIN-trained-Net and human data taken from Geirhos et al. (2019). Compared to S-Net, shape bias was increased most for B-Net, the second for B+S-Net, and the least for B2S-Net. However, the classification accuracy on the SIN dataset was significantly decreased for B-Net, only slightly for B+S-Net, and not at all for B2S-Net. Overall, among the four models, B+S-Net shows the most human-like performance. However, neither B-Net nor B+S-Net shows strong shape bias comparable to those of SIN-trained-Net and humans. These results indicate that while training with blurred images slightly increases the shape bias in comparison with training only with sharp images, blur training alone is insufficient to bring the bias closer to the human level.

### 3.3. Supplemental analyses

#### 3.3.1. Training schedule

We used the fixed schedule of learning rates as shown in Figure 1B. We determined the learning rate following a reference training script in torchvision library: https://github.com/pytorch/vision/tree/main/references/classification. To check the generality of our findings in particular about B2S-Net, we have additionally run a supplemental experiment to examine the effect of the training schedule. We trained B2S models while varying the initial learning rate and the number of epochs with discrete step sizes of [0.05, 0.01, 0.005, 0.001] and [60, 90, 120], respectively. The initial learning rate was reduced by a factor of 10 for every third of the total training epochs (as in the original experiment). The timing to decrease the sigma of the Gaussian kernel applied to the training images was also linearly extended (decreasing the sigma by 1 every 10, 15, and 20 epochs for the 60, 90, and 120 epoch training conditions, respectively).

As a result, we have obtained qualitatively similar amounts of blur robustness for all tested conditions except for a model with the initial learning rate = 0.05 and with training epochs = 120, in which the training diverged due to too large initial learning rate. All the models trained with learning rates 0.01 and 0.005 showed blur robustness/accuracy equivalent to the original B2S model regardless of the training epochs. We did not find any model that significantly outperformed the blur robustness of the original B2S model.

#### 3.3.2. B+S-Net with randomly varying blur strength

Considering that B2S-Net simulates human visual experiences during development, one can also consider that B+S-Net simulates human visual experience in everyday life where blurred images are occasionally mixed with sharp images due to image focusing errors. In the analysis so far, we have fixed the strength of image blur applied to training images for B+S-Net at σ = 4. Here, we trained a 16-class B+S-Net while randomly varying σ (0 px–4 px) to simulate our daily visual experience more realistically, and measured its performance on the (A)low-pass images, (B) jumbled/occluded images, and (C) shape-texture-cue-conflict images.

The results (Supplementary Figure S1) showed no significant changes in the performance on any of the test sets from the original B+S-Net. Fixing the blur strength is not the reason why blur learning is limited in its ability to reproduce human-like global object recognition.

#### 3.3.3. 1000-class-AlexNet

The analysis so far has been based on the 16-class-AlexNet. One may consider that 16 object classes are unrealistically small to simulate human object recognition. To address this concern, we also trained our networks with a 1000-class classification task (1000-class-AlexNet). For comparison with the main results, we used the 16-class-ImageNet to test performances,^3^ by mapping the output of the 1000-class-AlexNet into 16 classes based on WordNet hierarchy (Miller, 1995) using the mapping function described in Geirhos et al. (2018).

Concerning the accuracy for blurred images (Supplementary Figure S2A), the 1000-class-AlexNet exhibited an overall trend similar to that of the 16-class-AlexNet. However, we also found that the generalization effect of blur training beyond the blur strength used in training was smaller for the 1000-class-AlexNet than that for the 16-class-AlexNet. B-Net was firmly tuned to the blur strength used in training (σ = 4) and was barely able to recognize clear images. B+S-Net also showed a narrower blur tuning. B2S-Net showed no advantage over S-Net. Concerning the performances on the band-pass-filtered test images (Supplementary Figure S2B), the results of the 1000-class-AlexNet showed a similar trend to the 16-class-AlexNet. The effective bandwidth was somewhat narrower in the 1000-class version. It should be also noted that the 1000-class-AlexNet trained on Stylyzed-ImageNet (Geirhos et al., 2018) showed a human-like performance for band-pass test images. The results of the shape bias using the cue conflict images (Supplementary Figure S2D) show that there was little effect of blur training on shape bias when the 1000-class dataset was used. However, it should also be noted that the accuracy of the 1000-class models for the cue conflict images themselves was very low, meaning that the models were barely able to classify the test images to either the correct shape or texture label in the first place.

To conclude, we found no evidence supporting the idea that increasing the number of training categories makes blur training more effective in reproducing human-like robust object recognition.

#### 3.3.4. VOneNet (16-class)

VOneNet is a model in which the first layer of the 16-class-AlexNet is replaced with a VOneBlock (Dapello et al., 2020). The VOneBlock is a computational model that simulates the visual information processing in the V1 cortex of the brain, such as the response properties of simple cells and complex cells. It also simulates the stochasticity in neural responses by introducing noise. Importantly, multiscale Gabor filters tuned to low to high spatial frequencies are hard-coded in the VOneBlock.

One possible reason for the limited effect of blur training in reproducing human-like robust object recognition is that the training cannot produce human-like multi-scale filters in the early processing stage. If this were the case, through blur training, the model with VOneBlock would be able to achieve stronger robustness to low-pass and band-pass filtered images and stronger sensitivity to global configurations.

**Figure 3:**
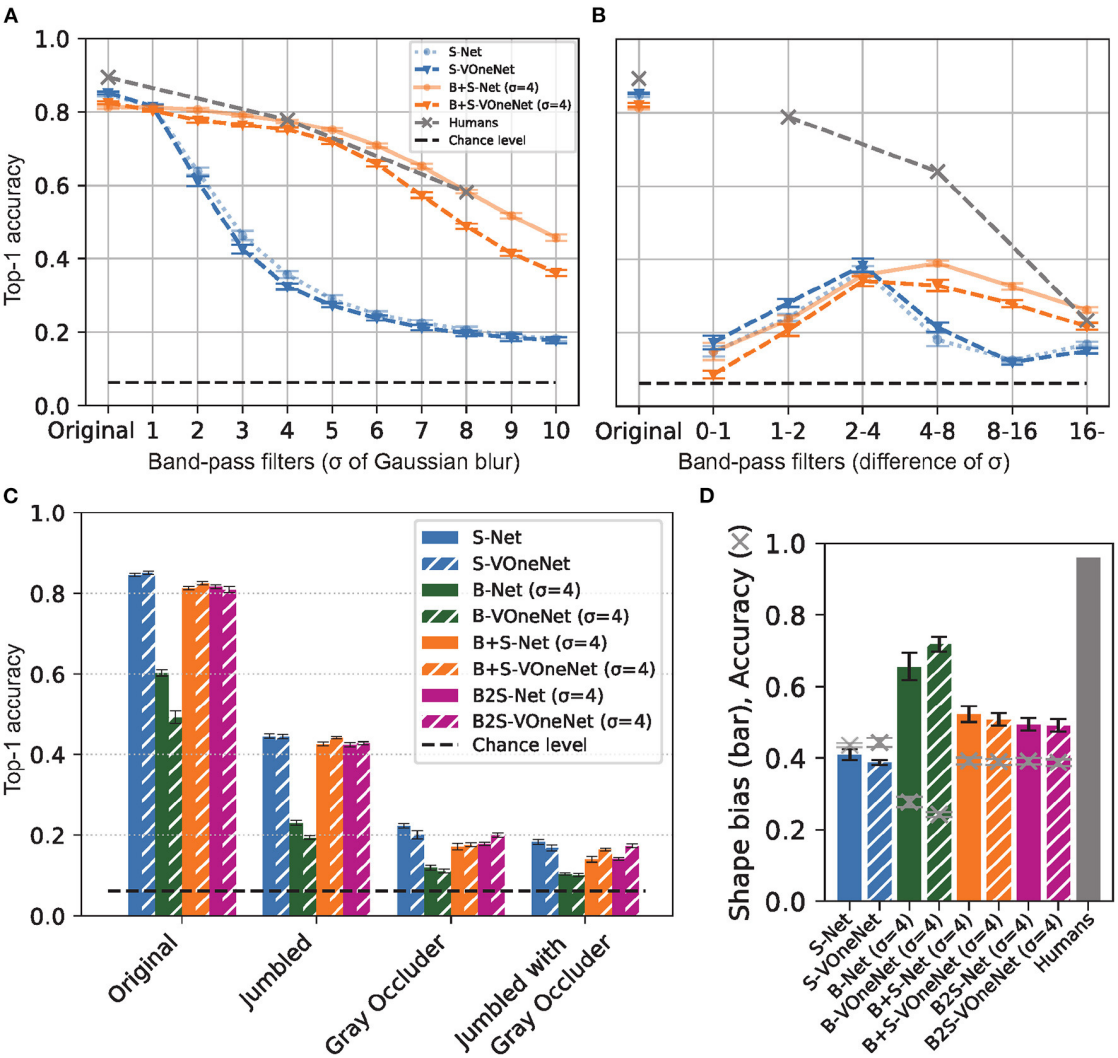
VOneNet with blur training. **(A)** Low-pass filtered object image recognition. **(B)** band-pass filtered object image recognition, **(C)** jumbled/occluded image recognition, and **(D)** shape-texture-cue-conflict image recognition. Although VOneNet has fixed V1-like first-stage mechanisms, the effects of blur training are similar to those of AlexNet. Error bars represent 95% confidence intervals estimated from the performances of eight models trained with different random seeds.

Contrary to this expectation, the introduction of the VOneBlock did not change the performance significantly. As shown in Figure 3, the results for each test set showed a remarkable degree of similarity between the models with and without VOneBlock. Thus, changing the lower-level layer to a model closer to the visual cortex did not affect the effects of blur training in terms of frequency and shape recognition.

#### 3.3.5. VGG16, ResNet50

Finally, we examine the performance of different network architectures other than AlexNet. The networks studied here are VGG16 (Simonyan and Zisserman, 2015) and ResNet50 (He et al., 2016). In general, the results were similar to those obtained with AlexNet, while the performance tended to be more tuned to trained blur strength (Supplementary Figures S3, S4).

## 4. Analysis of the internal representation of B+S Net

Thus far, we have analyzed the effect of training with blurred images on the basis of recognition performance, and found that the recognition performance of B+S-Net for low spatial frequency images is similar to that of humans. We have focused on the behavioral similarity/dissimilarity between humans and neural nets, leaving the internal processing of the B+S Net as a black box. In this section, we attempt to understand how B+S-Net acquires blur robustness similar to humans by analyzing internal representation analysis. The question is whether B+S-Net processes sharp and blurry images in a way computationally similar to the human visual system.

In general, when a visual processing mechanism is able to recognize both sharp and blurry images, we believe the way the image signals are processed inside the system can be roughly categorized into two cases.

**Figure 4:**
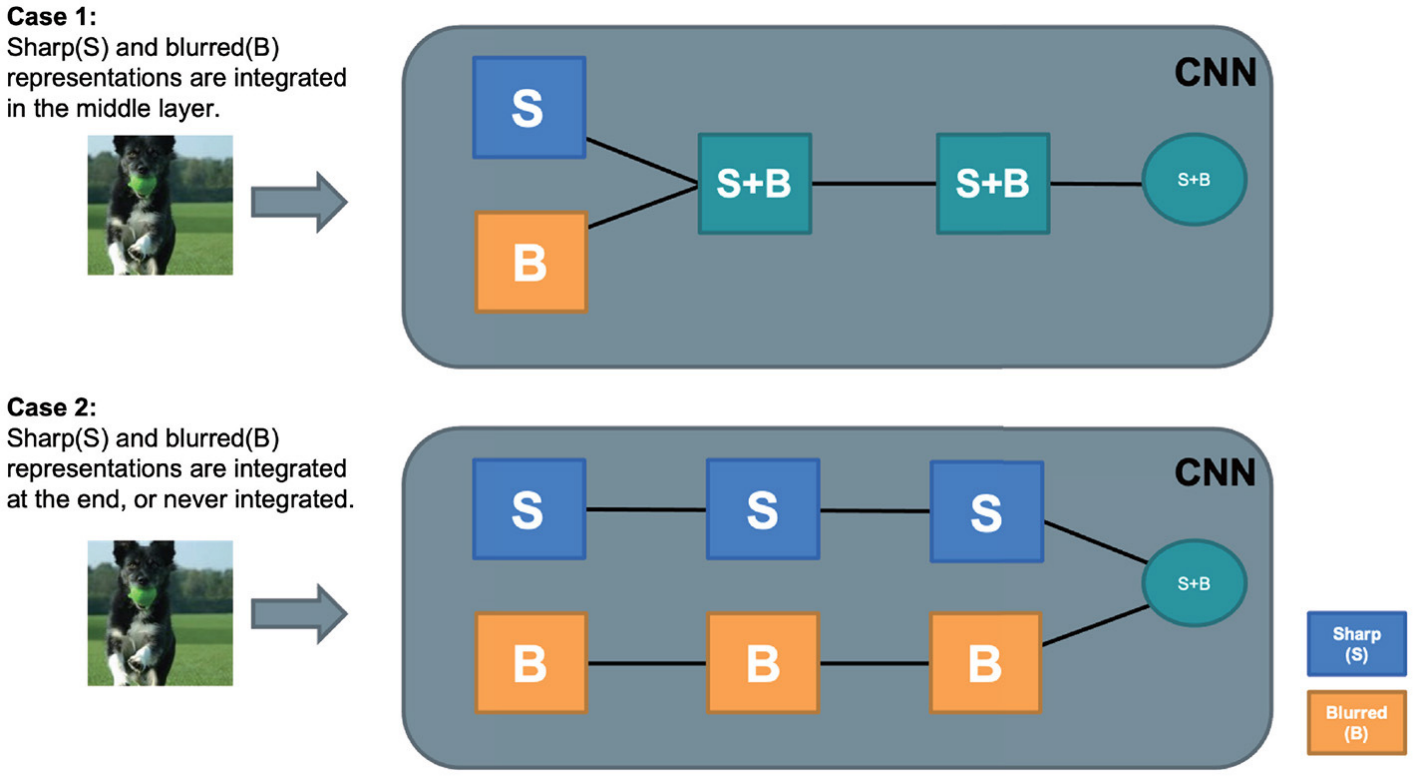
Two hypotheses about intermediate feature representation of B+S net. Case 1 is more efficient and presumably close to human processing.

- **Case 1:** Sharp and blurry images are processed by a common general process. Representations for sharp and blurry images are integrated into a common feature representation in the early to middle stage of visual processing. The following information processing is shared (Figure 4, top).
- **Case 2:** The sharp and blurred image features are processed separately by stimulus-specific processes until the outputs of the separate processes are integrated at the last stage to recognize the object (Figure 4, bottom).

Although we know no direct empirical evidence, it is likely that the structure of the human visual system for blurry image recognition is closer to Case 1 than to Case 2. This is because computational resource is more efficiently used in Case 1 than in Case 2. Considering that the human visual system has to cope with a wide range of image deformation other than image blur, having an efficient processing structure with a common higher stage must be a reasonable choice. On the other hand, CNNs with powerful learning abilities may create a specialized sub-network, each processing blurred images and sharp images separately, to optimize performance. B+S-Net could be a hybrid of B-Net and S-Net with little interactions between them. When using CNNs as a computational tool to understand human-like robust processing, we should check whether the processing strategy CNNs use to achieve blur robustness is not dissimilar to that of humans. If it were found to be dissimilar, we could learn little about the internal processing of the human visual system from this research strategy.

### 4.1. Receptive fields in the first layer

First, we visualized the receptive field of the first convolutional layer (Supplementary Figure S5) as was done in previous studies (Vogelsang et al., 2018; Jang and Tong, 2021). Some receptive fields look similar to those found in the early visual cortex. It appears that training method slightly alters the receptive fields. B+S-Net shows a shift of the spatial frequency tuning to the lower frequency compared to S-Net. In other words, B+S-Net is more sensitive to low-frequency information in the first layer.

Low-pass filtering is one way to make the internal representations similar between sharp and blurry images. How much the change in the spatial frequency tuning affects the representational similarity for sharp and blurry images in the first layer will be quantitatively evaluated in the next section.

When 1000-class-AlexNet is compared with 16-class-AlexNet, features with higher spatial frequencies are extracted. This may be because 1000-class-AlexNet needed to extract finer local features to perform more fine-grained classification. This tuning difference may explain why 1000-class-AlexNet shows weaker blur robustness than 16-class-AlexNet.

### 4.2. Correlation of activity in the intermediate layers

To analyze how the sharp and blurred image features are processed in each layer of the CNN models, we computed the average correlations of unit activities in each intermediate layer between the sharp and blurred image inputs (S-B correlation). The more representations and processing shared between clear and blurred images, the higher will be the activity correlation within the layer. Here, we calculated the S-B correlations in the following three cases: (1) the sharp and blurry image pair is generated from the same image, (2) different images from the same class, and (3) different images from different classes. For each case, we computed correlations for all possible sharp-blurry image pairs from 1,600 test images of the 16-class ImageNet. Then, the correlations were averaged across image pairs. The unit activities after the ReLU activation function were used to compute the correlations. (1) is for evaluating the representational similarity at the image level, while (2) and (3) are for evaluating the representational similarity at the category level. By comparing these three, we can infer both representational similarities and the corresponding processing stages.

**Figure 5:**
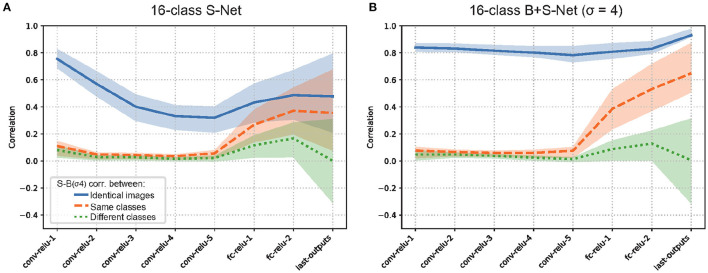
Representational similarity of sharp (unblurred) and blurred image inputs (S-B correlation) for S-Net **(A)** and B+S Net **(B)**. Blue: Pearson correlation in unit activity between sharp and blurred versions of the identical images. Orange: Correlation between sharp and blurred versions of different images of the same object class. Green: Correlation between sharp and blurred versions of different images of different object classes. The average correlation of the units in the layer with the interquartile range (25%-75%) is shown. 16-Class AlexNet. The results are consistent with Case 1.

Figure 5 presents the S-B correlation in each layer of 16-class S-AlexNet (left) and B+S-AlexNet (right).

In the initial layer (Conv1), while S-B correlations are close to zero when different images of the same class (the broken orange line) or different classes (the dotted green line) are used, they are high when the sharp-blurry image pairs from identical images are used (the solid blue line). When S-Net and B+S-Net are compared, B-S correlations are slightly higher for B+S-Net (0.84) than for S-Net (0.76). This agrees with the change in spatial frequency characteristics of the receptive filed we observed in the last section. If low-pass filtering in the first layer were powerful enough to completely remove the difference between sharp and blurry images, the correlation would be one.

In the subsequent convolutional layers, S-B correlations remain to be close to zero when different images of the same class or a different class are used. In S-Net, S-B correlation for the same images gradually drops as the layer goes. This suggests that these layers reduce the representational similarity between sharp and blurry images by extracting fine-scale image features only available in sharp images. On the other hand, in B+S-Net, S-B correlation for the same image remains high. This suggests that these layers extract robust image features commonly available in sharp and blurry images, supporting the idea that B+S-Net achieves blur-robust recognition by forming a common internal processing structure consistent with Case 1.

In the final full-connection layers, S-B correlations gradually increase for the same image and for the same class, while increasing and then dropping for the different class. The pattern of change is similar for S-Net and B+S-Net, but the correlations for the same image/class are higher for B+S-Net, in agreement with the higher classification accuracy of B+S-Net for both sharp and blurry images.

**Figure 6:**
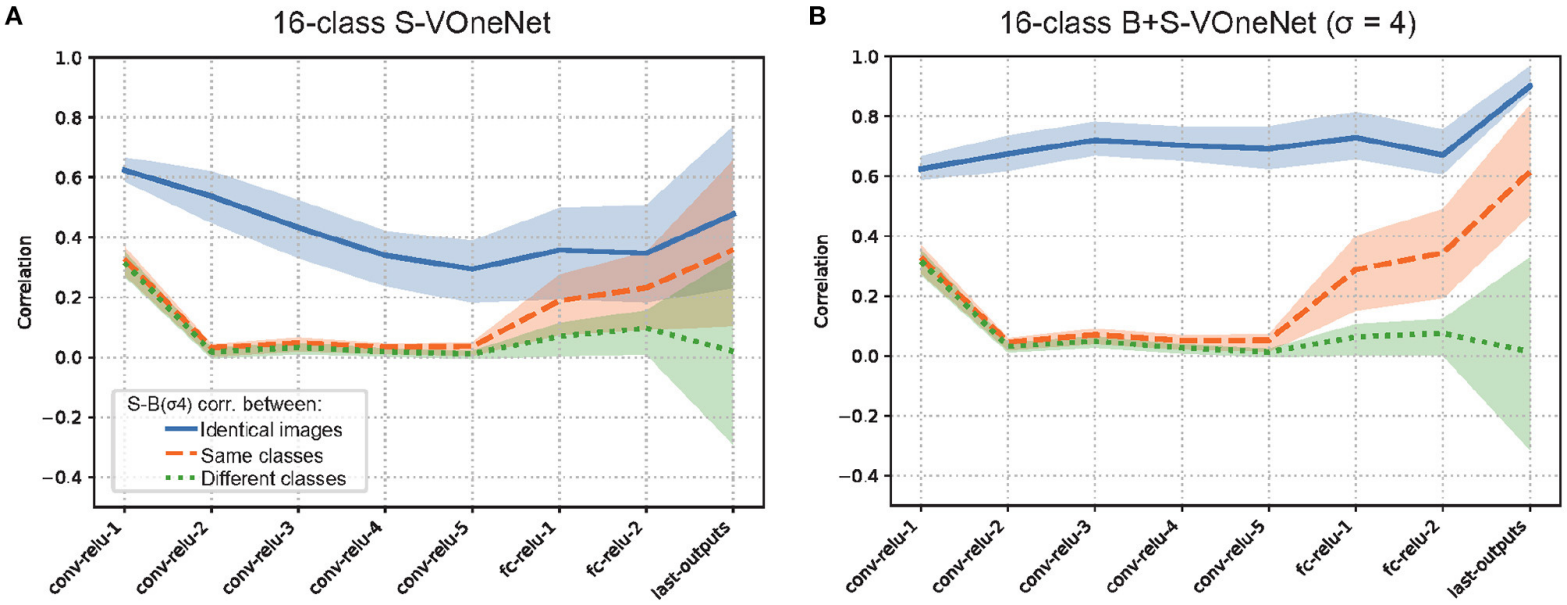
Representational similarity of sharp (unblurred) and blurred image inputs for S-Net **(A)** and B+S Net **(B)**. VOneNet. The pattern of results is similar to Figure 5.

To see the generality of our finding, we also applied the same analysis to 1000-class AlexNet (Supplementary Figure S6) and 16-class VOneNet (Figure 6). In general, the patterns of B-S correlations for both are similar to that we found for 16-class AlexNet, but two issues are worth mentioning. First, S-B correlation in the first convolutional layer is lower for 1000-class AlexNet than for 16-class AlexNet (0.65 for S-Net and 0.69 for B+S-Net), in agreement with the higher-frequency preference of the initial receptive fields for 1000-class AlexNet (Supplementary Figure S5). Second, for VOneNet in which the first layer is hard-coded as a Gabor filter bank, while S-B correlation in the first convolutional layer is the same for S-Net and B+S-Net, S-B correlation of B+S-Net elevates in the subsequent layers. This indicates that B+S-Net forms the features common to both sharp and blurred images from the multiband information extracted in the first layer. The initial low-pass filtering is effective, but not necessary for B+S-Net to achieve blur-robust object recognition.

### 4.3. Visualization of the internal representations by t-SNE

To understand how the sharp and blurry images are represented in the intermediate layers of the CNN models, we also attempted to visualize them using the dimensionality reduction algorithm, t-SNE (van der Maaten and Hinton, 2008). Specifically, we recorded the activities of each layer obtained from sharp and blurry images and compressed them into two dimensions for visualization. The two input parameters of t-SNE, perplexity and iteration, were set to 30 and 1000, respectively. The results shown here are visualizations of 10 pairs of sharp and blurred images of the same image sampled for each of the 16 classes.

The visualizations of the intermediate layer activities for the sharp and blurred images are shown in Supplementary Figures S7, S8. First, in early convolutional layers of S-Net, the representations of the sharp and blurry images overlap, and those of the same class are scattered. As the layer goes deeper, the representations of the sharp and blurry are separated, and only sharp images of the same class are clustered. Blurry images remain scattered and separated from sharp images in the final output. Next, in early convolutional layers of B+S-Net, the representations of the sharp and blurry images overlap, and those of the same class are scattered, as in S-Net. As the layer goes deeper, however, representations of the sharp and blurry images do not separate, and both sharp and blurry images of the same class are clustered. These results agree with the trends indicated by the representational similarity analysis in the last section, providing further support of the idea that B+S-Net achieves blur-robust recognition by forming a common internal processing structure consistent with Case 1.

### 4.4. Generalization test using zero-shot transfer learning

The results of the S-B correlation analysis in sections 4.2 and 4.3 suggested that the representations are shared between the sharp and blurry versions of the same images in the intermediate layers of B+S-Net. This suggests that the intermediate layers of B+S-Net may have the ability to extract robust image features effective in recognizing both sharp and blurry images. One way to test this idea is to see whether the B+S training extends its effect beyond the image classes used in training, since general robust features should be useful in general.

Using zero-shot learning, we examined the generalizability of the shared representation acquired by blur training to the unseen classes. We trained a subset of object classes, either one or eight in 16 classes, without using blurry images while training the remaining classes using both blurry (σ = 4) and sharp images, and later evaluated the classification accuracy for that subset of classes using blurry images. Conversely, we also trained a subset of classes without using sharp images while training the other classes using both blurry and sharp images, and later evaluated the classification accuracy using sharp images. Therefore, there were in total four conditions, i.e., training without blurry or sharp images for one or eight classes (w/o 1/16B, w/o 8/16B, w/o 1/16S, w/o 8/16S). We used the 16-class AlexNet for this test.

The recognition accuracy for the unseen image types (either blurry or sharp) in the four test conditions is shown in Table 2.

**Table 2:** The results of zero-short learning test.

**Training method**	**Unseen labels**	**Seen labels**
Training without B images for one class (w/o 1/16B)	0.19	0.78
Training without B images for half of the classes (w/o 8/16B)	0.05	0.87
Training without S images for one class (w/o 1/16S)	0.15	0.81
Training without S images for half of the classes (w/o 8/16S)	0.03	0.90

Classification accuracy for B+S-Net trained without using blurred or sharp images for specific object classes. Chance level accuracy = $0.0625\left(=\frac{1}{16}\right)$.

When either blurry or sharp images were excluded for half of the training classes, the models were not able to recognize these classes of images with the unseen image type. On the other hand, when either blurry or sharp images were excluded for one training class, the accuracy for the unseen class is about three times the chance level ($\frac{1}{16}=0.0625$). Therefore, although the effect of the generalization of the sharp and blurry features to unseen categories was limited in terms of the zero-shot transfer performance, some amount of transfer was clearly observed at least when there was only one excluded class.

**Figure 7:**
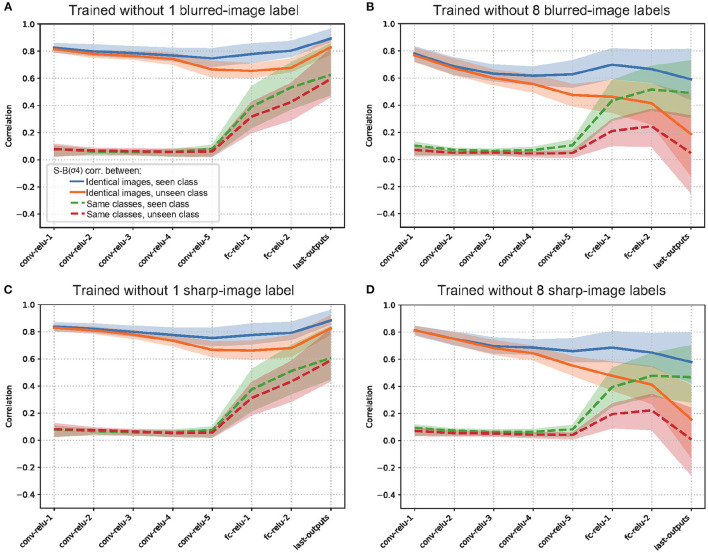
Representational similarity analysis for zero-shot learning test when **(A)** one blurred-image label, **(B)** eight blurred-image labels, **(C)** one sharp-image label, or **(D)** eight sharp-image labels is/are excluded from the training set. Test set was blurred images for **(A, B)**, and sharp images for **(C, D)**. The results suggest the shared representation acquired by blur training can be reused, at least partially, to recognize an unseen object class.

To further analyze the internal representations of the models trained in the transfer experiment, we examined the S-B correlations in the intermediate layers of each model. When either blurry or sharp images were excluded for half of the training classes (Figures 7B, D), the S-B correlation from identical images is significantly reduced in the middle to high layers for the unseen category (orange line), compared to that for the seen category (blue line). On the other hand, when either blurry or sharp images were excluded for one of the training classes (Figures 7A, C), the S-B correlation from identical images for the unseen category (orange line) remains almost as high, albeit slightly lower than that for the seen category (blue line). Therefore, although the shared representation for the blurry and sharp images did not seem to generalize well to the unseen class in terms of the performance level, the similarity of the internal representations appeared to be high between the seen and unseen classes for the model with one excluded class. The reason for this apparent discrepancy is presumably because the misclassification to a class with a similar representation was induced by the imperfect alignment of blur-sharp representations. In fact, a confusion matrix (Supplementary Figure S9) indicates that the misclassifications in the model with one excluded class were mostly from “No.15: truck" class to “No:6 car" class.

Overall, the zero-shot transfer analysis suggested that the shared representation acquired by blur training can be reused, at least partially, to recognize an object class with an unseen image type (either blurry or sharp) during training. This further supported the view that common representations that are invariant to blurry and sharp image inputs are formed in the early and middle stages of visual processing by blur training (Case 1 in Figure 4). In a similar way, humans might efficiently acquire blur robust representations to general object categories just by being exposed to blurry images of a limited number of objects.

### 4.5. Internal representations for high-pass and low-pass images

We have analyzed the internal representation of B+S-Net for sharp and blurry images, and found B+S-Net has an efficient human-like processing mechanism at least for these images. However, we have also shown in section 3 that B+S-Net does not behave similarly to humans in object recognition for other modified images including high-pass filtered images. To further evaluate B+S-Net as a computational model of the human visual system, we analyzed its internal representation for high-pass and low-pass (blurry) images. A recent human fMRI study (Vaziri-Pashkam et al., 2019) suggests that the representations for high-pass and low-pass images of the same object category are segregated in V1, while integrated and clustered in the higher visual areas.

To investigate how the high- and low-frequency information is represented in S-Net and B+S-Net, we visualized the activity in the intermediate layers for 10 pairs of high-pass (H, σ1−σ2) and low-pass (L, σ = 4) versions of the same image using the t-SNE (van der Maaten and Hinton, 2008) (perplexity = 30, iteration = 1,000). The visualization results (Supplementary Figures S10, S11) show that the representations of high-pass and low-pass images are less segregated in B+S-Net than in S-Net. We cannot find class-based clustering of high-pass and low-pass images in higher layers of either S-Net or B+S-Net, in agreement with our finding in section 3 that neither S-Net nor B+S-Net can recognize objects in high-pass images, but in disagreement with the representation in human visual cortex (Vaziri-Pashkam et al., 2019).

**Figure 8:**
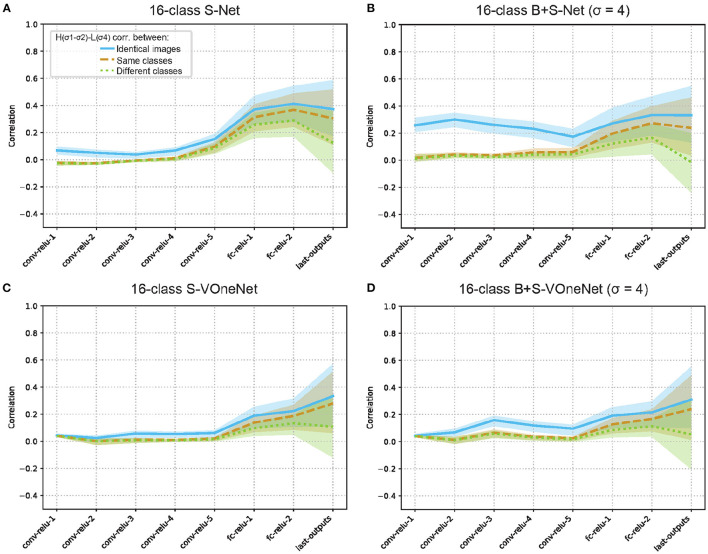
Representational similarity of low-pass and high-pass image inputs for S-Net **(A, C)** and B+S Net **(B, D)**. 16-Class AlexNet **(A, B)** and VOneNet **(C, D)**. There is no evidence that blur training facilitates high-level frequency integration as found in human visual cortex.

To further examine the representations for high-pass and low-pass images, we computed the average activity correlation (H-L correlation) of the middle layers of S-Net and B+S-Net between the high- and low-frequency images (Figure 8). In the convolutional layers, the H-L correlation was low even for the same image. Slightly higher correlations for B+S-Net than for S-Net suggest that early layers of B+S-Net have more broadband tuning. In the fully connected layers, the H-L correlation gradually increased. Although this is in line with class-based clustering of high-pass and low-pass images, the increasing trend was weak, and was not enhanced by blur training. The average same-class correlation did not exceed 0.3 for the final output of B+S-Net. In sum, there are significant differences in the internal representations for high-pass and low-pass images between B+S-Net and the human cortex, and there is no evidence that blur training facilitates high-level frequency integration.

To see the effect of initial layer on the representation of high-pass and low-pass images, we also analyzed the H-L correlation of VOneNet, which has fixed multi-scale Gabor filters in the first layer. The H-L correlation for the same images in the convolutional layers is low, and, again, there is no evidence of strong integration of low- and high-frequency information in higher layers, unlike representation in the human visual cortex (Vaziri-Pashkam et al., 2019).

## 5. General discussion

In this study, we investigated the effect of experiencing blurred images on forming a robust visual system to the environment as one of the factors for constructing an image-computable model of the human visual system. To this end, we compared the recognition performance of CNN models trained with a mixture of blurred images using several different strategies (blur training). The results show that B+S-Net trained with a mixture of sharp and blurred images is the most tolerant of a range of blur and the most human-like. In addition, training in the order from blurred to sharp images was not very beneficial. Other evaluations of the model's performance with test stimuli showed that blur training did not improve the recognition of global spatial shape information, or only slightly. The analysis of the internal representation suggests that B+S-Net extracts common features between sharp and blurred images. However, it does not show integration of multi-scale (high and low frequency) frequency information, unlike in the human visual cortex.

In section 3, we compared the effect of training with blurred images on the CNN models in terms of object recognition performance. In all the CNN models we tested, the recognition performance of low spatial frequency features was improved by blur training. In particular, the model trained simultaneously on blurred and sharp images (i.e., B+S-Net) showed blur robustness across a wide range of image blur close to that of humans.

On the other hand, the blur robustness of B2S-Net was weaker than that of B+S-Net. The models showed better performance when trained on blurred and sharp images simultaneously, rather than on a schedule that simulated human visual development. This result apparently disagrees with the study of Vogelsang et al. (2018), which showed that training in the order of low resolution to high resolution improved blur robustness of a CNN model in face recognition. This difference can be attributed to the difference in the task adopted in our study and Vogelsang et al.'s (i.e., general object classification vs face classification) (Jang and Tong, 2021). A recent study using object recognition (Avberšek et al., 2021) reports that the effect of training schedule is consistent with ours. The task difference may be related to the fact that the optimal discriminative features for object recognition are biased toward high frequencies while only low-frequency features are sufficient for good face classification accuracy (Jang and Tong, 2021).

The failure of B2S-Net to recognize blurry images indicates that simply simulating the development of visual acuity during training cannot account for the blur robustness of human vision in general object recognition. However, even after the completion of visual development, we still experience blurred retinal images on a daily basis due to defocus as well as motion blur, and scattering caused by climatic conditions such as rain and fog and by the transmission of translucent objects. In this sense, B+S Net, trained simultaneously with both blurry and sharp images, can be regarded as reproducing biologically plausible situations to some extent. In addition, the CNNs we tested do not include a mechanism that prevents the forgetting of previously learned representations. B2S-Net may have forgotten the processing for low-frequency components because it was trained only on sharp images in the last 20 epochs. Therefore, B2S-Net may be able to recognize blurred images as well as B+S-Net by adding a mechanism that prevents the model from forgetting the representations tuned for blurred images learned in the early phase of training. Machine learning literature has suggested a few methods to prevent so-called catastrophic forgetting in continual learning (de Melo et al., 2022). One is to protect the weights relevant to the stimuli learned in the early phases of training (Kirkpatrick et al., 2017). This is reminiscent of the critical period of biological neural networks, which strengthens the impact of early childhood experiences on development of the human visual system. Another method is to use the memory of relevant prior information to retrain the network with new information (Aljundi et al., 2019). This mechanism will make the effect of B2S training similar to that of B+S training. With such an additional mechanism against forgetting, B2S-Net may be able to show performance comparable to B+S-Net.

Our results also show that B+S Net has acquired human-level blur robustness but has not acquired human-like global visual processing. The performance test using band-pass filtered images showed that all CNN models, including B+S Net, were not good at utilizing band-limited features while humans retained good accuracy in the mid to high-frequency range. Although the shape bias of the blur-trained models was slightly enhanced, it was not enough to reach the human level. The test using the images with local occlusions revealed that all the models relied primarily on local features, did not utilize the global configuration, and were critically vulnerable to local occlusions. All these results are in stark contrast to human visual processing, which is known to rely more on global configural relationships and shape information and is less sensitive to partial occlusions in object recognition tasks. Therefore, our results indicate that the information processing learned in B+S-Net is still markedly different from that of the human visual system (Geirhos et al., 2021; Baker and Elder, 2022).

Our results reveal that what the networks cannot acquire from blur training is human-recognizable global configuration features present not only in sharp and blurry images but also in high-pass images and texture-shape cue conflict images. Note that the similarity of these classes of images is supported by a finding that the SIN-trained Net shows good recognition for high-pass images as well. In high-pass images, local edge features defined by high-frequency luminance modulations produce global configurations at a scale much larger than a fine-scale edge detector. For detection of these global features, second-order processing such as those modeled by an FRF (filter-rectify-filter) model for human vision [e.g., Graham and Landy (2004)] may be necessary. It seems that object recognition training with sharp and blurred images alone does not provide neural networks with the ability to process second-order features.

According to the comparison of the model architectures, there was no qualitative difference in the effect of blur training. Importantly, we found that VOneNet, which hard-coded the computational processes in the primary visual area (V1) in the front end of AlexNet, did not show improvement in any of the tasks tested in this study. This indicates the limited impact of the initial layer on the frequency tuning at the task performance level and on the mid to high-level information processing related to the shape bias and the configural effect. On the other hand, we also found a few notable differences in the frequency tuning patterns between the architectures. For example, the loss of blur robustness observed in B2S-Net was more prominent in 1000-class AlexNet as well as in 16-class VGG16 and 16-class ResNet50 than in 16-class AlexNet. B+S-Net and B-Net in these architectures were also more narrowly tuned to the blur strength used during training. For the 1000-class AlexNet, the reason for this may be attributed to the fact that the models were exposed to a higher number of images (and thus went through a higher number of weight updates) when using the 1000-class dataset than the 16-class dataset. For the 16-class VGG16 and 16-class ResNet50, differences in model architecture such as increased depth, reduced kernel size, and residual connections (in the case of ResNet) may have resulted in improved learning efficiency, thereby making them more likely to specialize in features that are optimal for the current blur strength. In addition, we also found that VGG16 demonstrated higher accuracy for the band-pass filtered images with high spatial frequency than the other architectures, though we have not yet been able to ascertain why.

In section 4, we analyzed how B+S-Net, which performed similarly to humans in a low spatial frequency image classification task, processed sharp and blurred images. The activity correlation between sharp and blurred images increased in B+S-Net. The results suggest that B+S-Net extracts more common features from sharp and blurred images than S-Net.

The results of zero-shot transfer learning support this view. While the generalization accuracy is not very high, the confusion matrix and the internal activity correlation suggest that B+S training produces a certain degree of common representation between blur and sharp features, which can be used even for unlearned categories.

These results suggest that B+S-Net recognizes sharp or blurred images using common representations, rather than using separate representations. The results also suggest that it is not only linear low-pass filtering in the first layer, but also a series of non-linear processing in the subsequent layers, that produces the common representations. In this respect, we may be able to get useful computational insights into human processing from the analysis of B+S-Net.

Whereas we found B+S training facilitates the development of common processing for sharp (broadband) and blurred (low-pass) images, we found little evidence for B+S training facilitating the development of common processing for low-pass and high-pass images, nor integration of sub-band information. These results suggest that the frequency processing by B+S-Net is critically different from that by the human visual cortex. How can we make the frequency processing more closely resemble that of the human visual system? Several machine learning techniques including data augmentation and contrastive learning may be used to force the network to integrate sub-band information. Note, however, that as a tool to understand human visual computation, it is important that the model training is natural and plausible for the development of the human visual system, like blur training.

In conclusion, training with blurred images provides performance and internal representation comparable to that of humans in recognizing low spatial frequency images. It does narrow, but only slightly, the gap with the human visual system in terms of global shape information processing and multi-scale frequency information integration.

## Data availability statement

The original contributions presented in the study are included in the article/Supplementary material, the data and codes that support the findings of this study are available at https://github.com/KUCognitiveInformaticsLab/blur-training.

## Ethics statement

The studies involving human participants were reviewed and approved by Research Ethics Committee at Graduate School of Informatics, Kyoto University. The patients/participants provided their written informed consent to participate in this study.

## Author contributions

SY performed the experiments and data analysis under the supervision of TF and SN, and drafted the first manuscript. All authors contributed to the experimental design, manuscript writing, and approved the submitted manuscript.

## Footnotes

^1^The equation is implemented in OpenCV's GaussianBlur function that we used to apply Gaussian filtering. In this function, the kernel size was adaptively determined from the size of sigma.

^2^2]Texture-shape cue conflict image: taken from the GitHub page of Geirhos et al. (2019): https://github.com/rgeirhos/texture-vs-shape/tree/master/stimuli/style-transfer-preprocessed-512, reference date: 2021/07/26.

^3^3]The models trained on the 1000-class dataset were directly used in the analysis. Fine-tuning the 16-class ImageNet may yield different results.
