# Supplementary material for: Does training with blurred images bring convolutional neural networks closer to humans with respect to robust object recognition and internal representations?
Source: Front Psychol. 2023 Feb 15;14:1047694. doi: 10.3389/fpsyg.2023.1047694 (PMC9975555; doi:10.3389/fpsyg.2023.1047694)
Supplement: Supplementary file 1 [file Presentation_1.pdf]

# Supplementary Material

## 1 SUPPLEMENTARY FIGURES

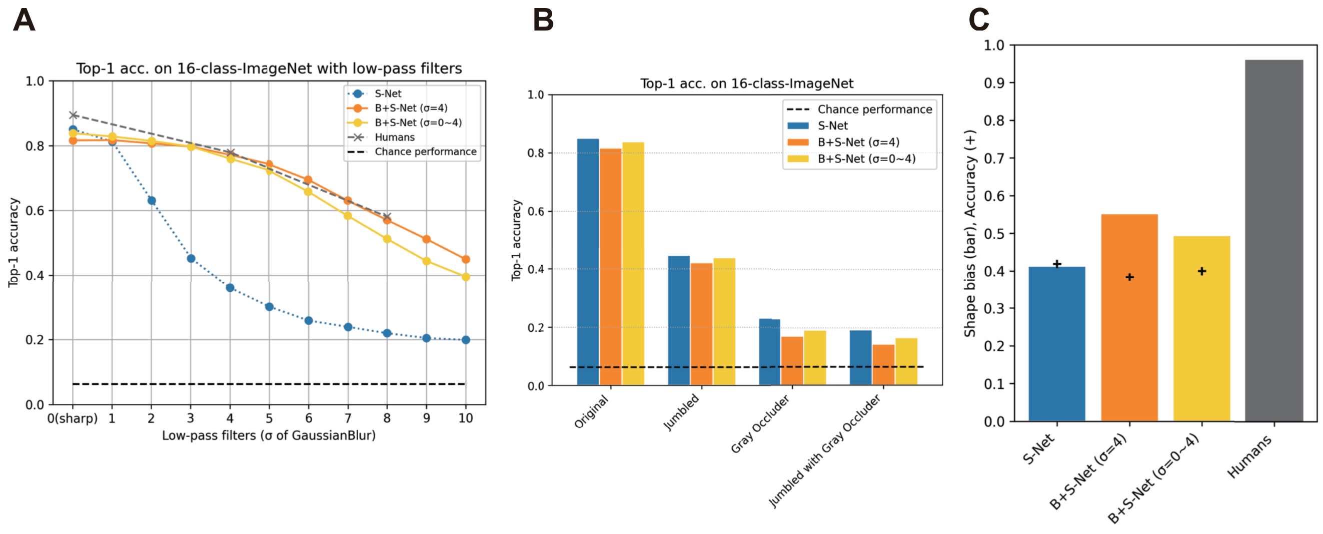


Figure S1: Comparison of B+S Net performance when the standard deviation ($\sigma$) of the Gaussian blur kernel of the training image is fixed at 4 px or varied from 0 px to 4 px. Recognition performances of (A) low-pass images, (B) jumbled/occluded images, and (C) shape-texture-cue-conflict images. We found no significant changes in the performance on any of the test sets from the original B+S-Net. Fixing the blur strength is not the reason why blur training is limited in its ability to reproduce human-like global object recognition.

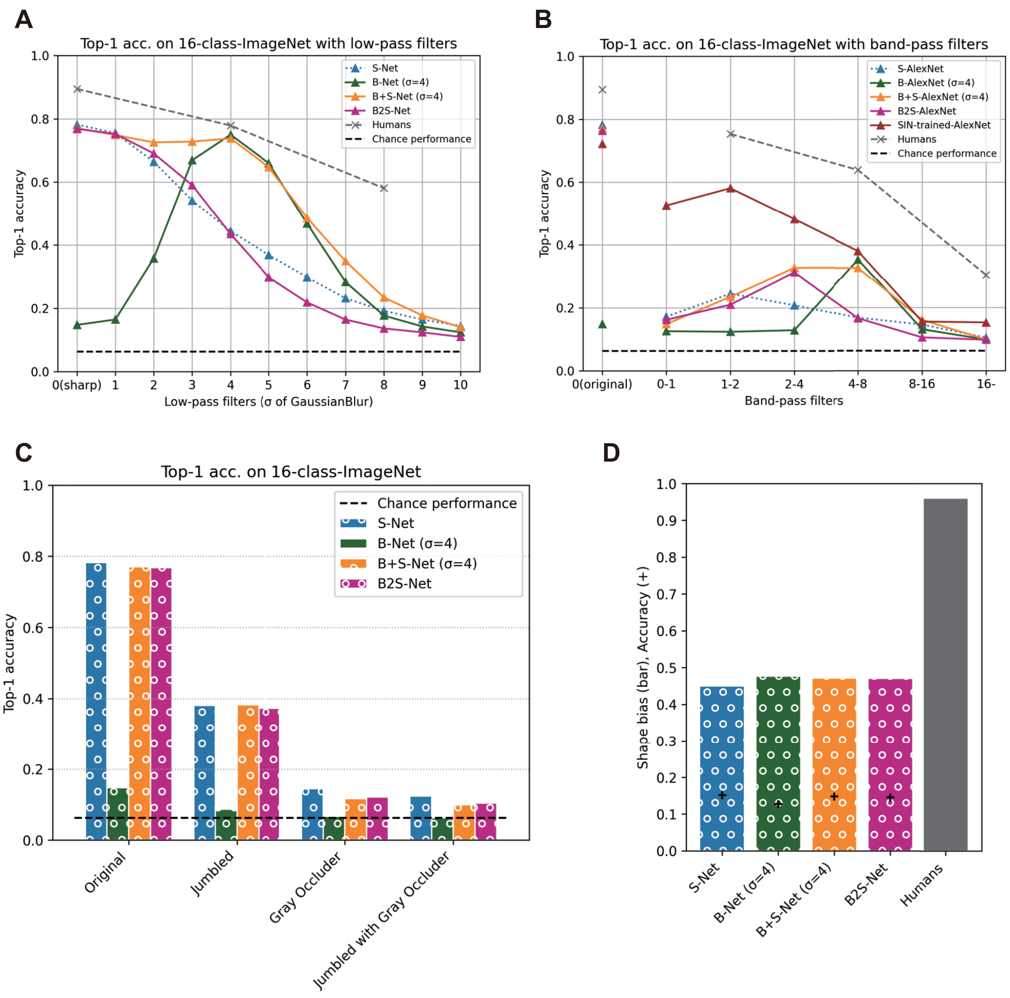


Figure S2: 1000 class AlexNet with blur training. S-Net, B-Net, B+S-Net, and B2S-Net were trained on the ILSVRC2012 ImageNet (1000-class classification task, 1.2 million training images) from scratch. Otherwise, we used the same training procedure as the main study. The 16-class-ImageNet was used to test performance. (A) Accuracy for blurred images. The overall results of the 1000-class-AlexNet exhibited a shared trend with those of the 16-class-AlexNet. B+S-Net showed blur robustness to a broader range of test blur strengths than S-Net while B-Net showed robustness only around the blur level it was trained with. B2S-Net did not show any improvement over S-Net, probably due to forgetting in the last 20 epochs during which the model was trained with only sharp images. However, we also found that the generalization effect of blur training beyond the blur strength used in training was smaller for the 1000-class-AlexNet than that for the 16-class-AlexNet. B-Net was firmly tuned to the blur strength used in training ($\sigma = 4$) and was barely able to recognize clear images. (B) Accuracy of the band-pass-filtered test images. Again, the results of the 1000-class-AlexNet showed a similar trend to the 16-class-AlexNet but the effective bandwidth was narrower in the 1000-class version. In addition, as with the 16-class-AlexNet, the accuracy for high-frequency images was low, indicating that the 1000-class models could not recognize the information composed only of high-frequency patterns. (C) Effect of global configuration of local patches on the performances of 1000-class AlexNet. We found that the trend was exactly the same as observed with the 16-class version: high accuracy (about 40%) for *Jumbled* images and significantly worse accuracy for *Gray Occluder* images. In other words, the CNNs could classify images to some extent using local information alone, but it was challenging for them to globally integrate the information for object recognition. (D)Shape bias using the cue conflict images. There was little effect of blur training on shape bias when the 1000-class dataset was used. However, it should also be noted that the accuracy of the 1000-class models for the cue conflict images themselves was very low, meaning that the models were barely able to classify the test images to either the correct shape or texture label in the first place.

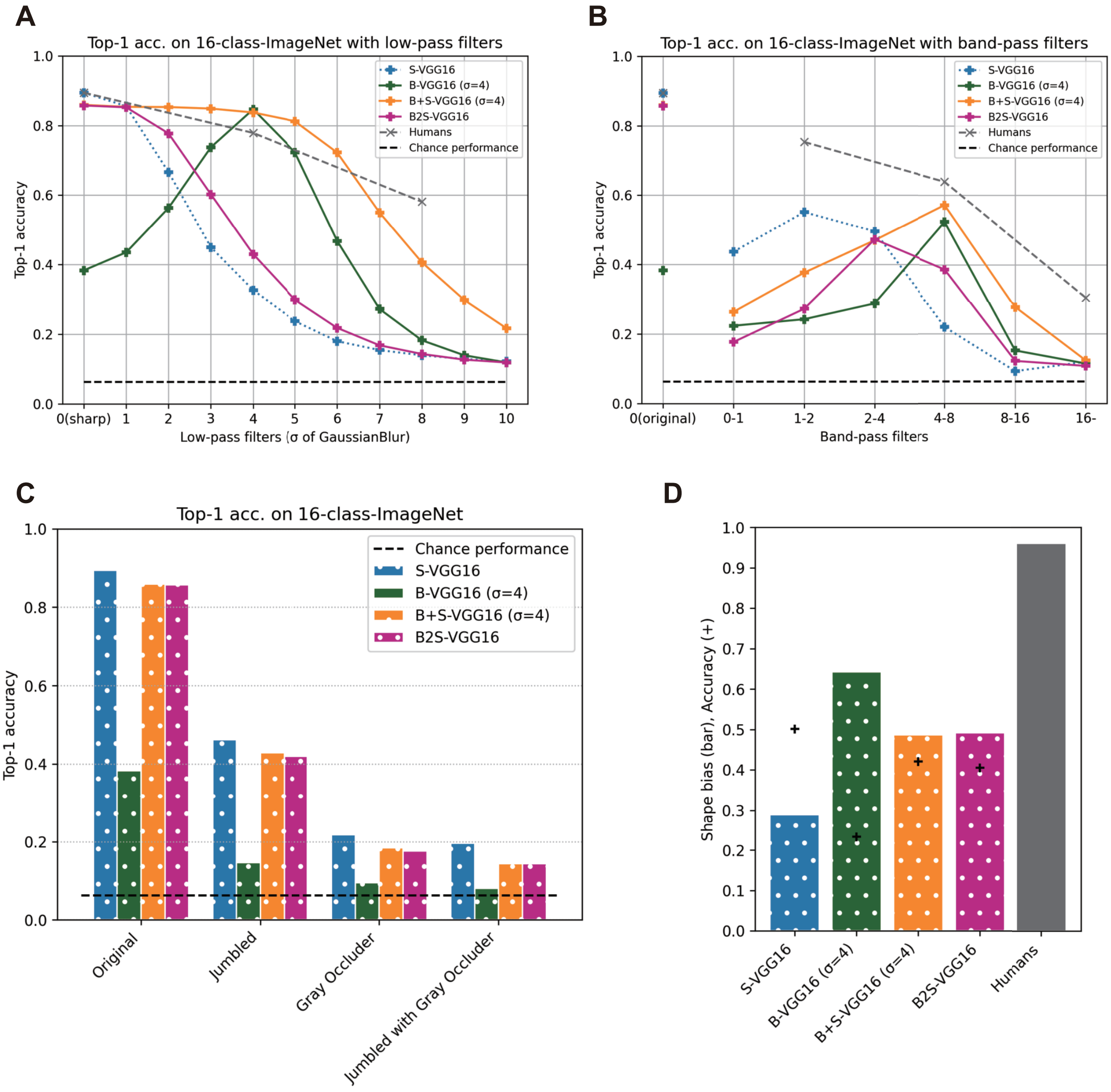


Figure S3: Different network architectures other than AlexNet. Here are the results for VGG16 with blur training, and the next figure shows the results for ResNet50 with blur training. We set the final output of all networks to 16 classes. Note that we explicitly indicate the name of each network along with the training procedure in this caption (e.g. S-AlexNet for S-Net with AlexNet architecture). (A) Blur image test. The overall trend of results for ResNet50 (Fig. S3A) and VGG16 (Fig. S4A) is similar to that for AlexNet. However, compared to B-AlexNet, B-VGG16 and B-ResNet50 displayed a tendency to overfit to the blur strength used during training (i.e., $\sigma = 4$). Although B+S-VGG16 and B+S-ResNet50 outperformed humans on some low-frequency images, the accuracy dropped sharply around $\sigma = 6$ and fell below humans at $\sigma = 8$. In this respect, B+S-AlexNet appears to have the most human-like performance and blur robustness among the three architectures. (B) Band-pass image test. Although variation in tuning patterns due to training methods was similar across architectures, we found that VGG16 (in particular, S-VGG16) was better at recognizing objects using high-frequency components than the other architectures. Training with blurred images decreased the reliance on high-frequency components in VGG16. (C) Local patch jumbling/occlusion test. We found no significant difference from AlexNet. Regardless of the architecture, the CNN models tended to rely on local information rather than global configural information for object recognition. (D) The shape bias was lower (the texture bias was higher) for S-VGG16 and S-ResNet50 than for S-AlexNet. This result may be related to the fact that S-VGG16 and S-ResNet50 had higher accuracy for high-frequency images than S-AlexNet. Thus, decreasing the reliance on high-frequency components might have some effect on increasing the shape bias. However, the increase in shape bias due to blur training was not enough to reach the human level in any of the three architectures.

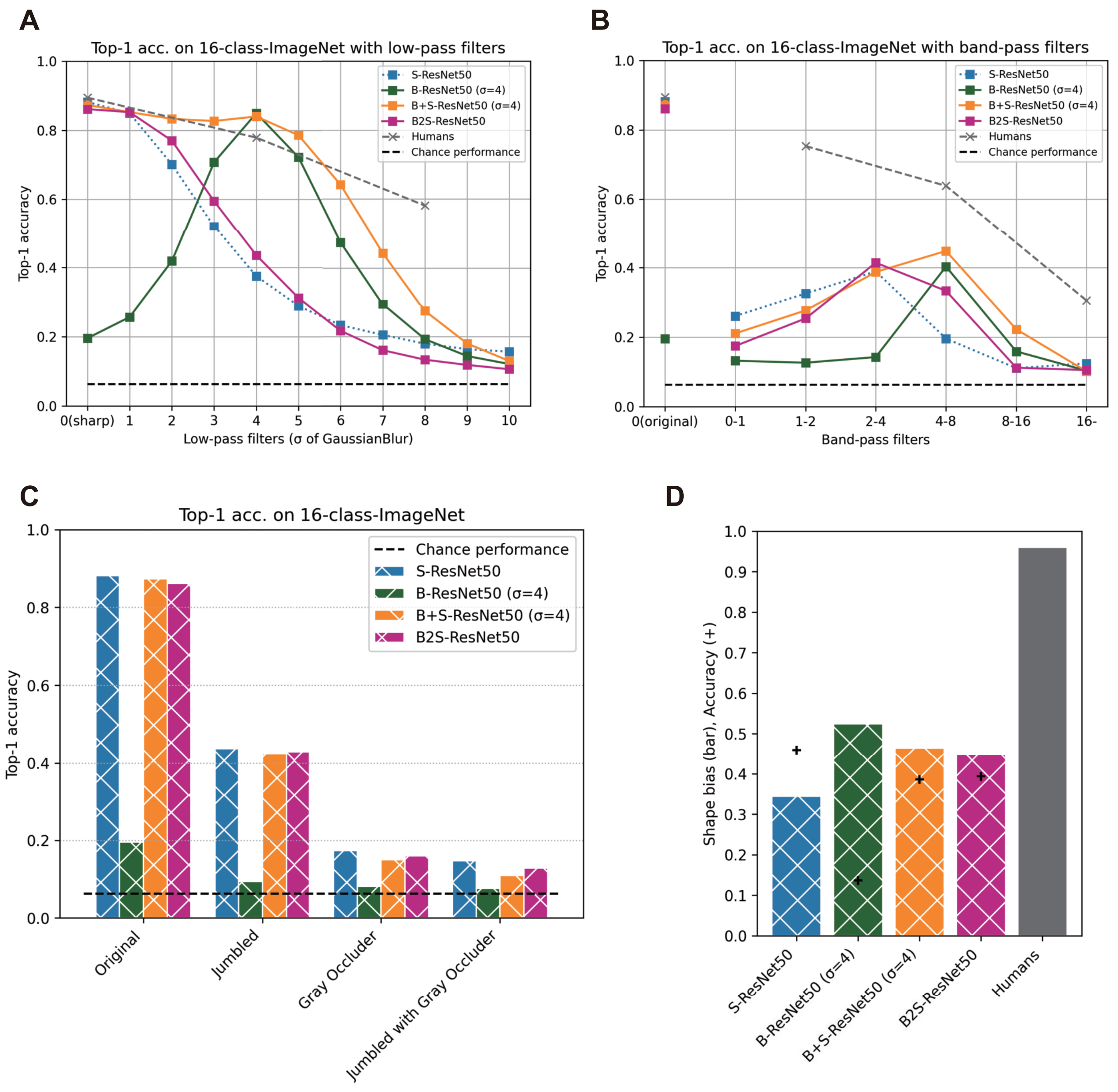


Figure S4: ResNet50 with blur training. See Caption of Figure S3.

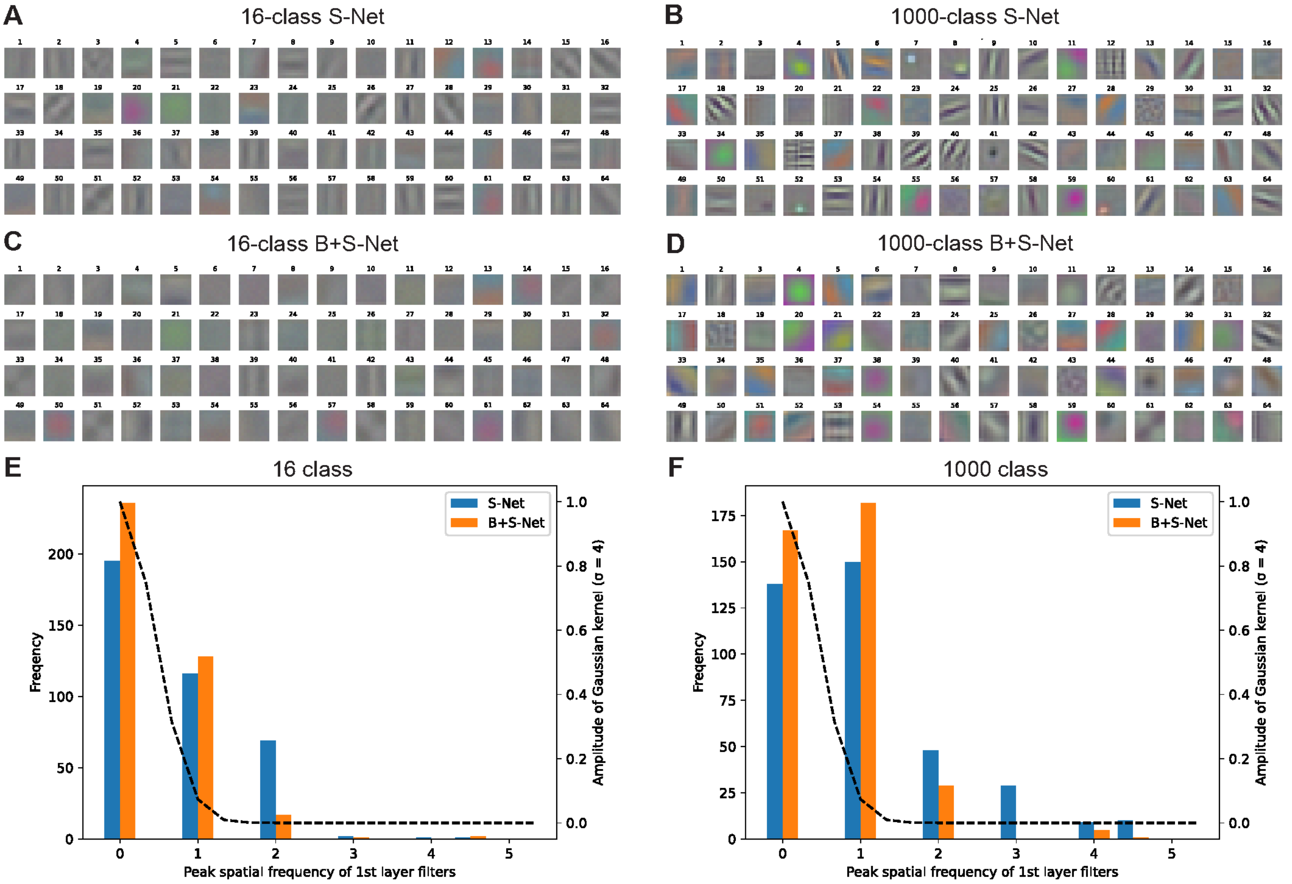


Figure S5: Visualization of the receptive fields of the first convolutional layer. (A)-(D) The visualizations of 16-class S-Net, 1000-class S-Net, 16-class B+S-Net, and 1000-class B+S-Net. (E) Comparison of histograms of preferred spatial frequencies between 16-class S-Net and 16-class B+S-Net. (F) Comparison between 1000-class S-Net and 1000-class B+S-Net. The preferred frequency was computed as the peak frequency of the Fourier transform for each RGB channel of each filter. The peaks of both horizontal and vertical frequencies were concatenated to obtain the histogram. The frequency is expressed as cycles per filter size (11 pixels). The broken line indicates the amplitude spectrum of the Gaussian blur kernel used for training the B+S Net ($\sigma = 4$). The units prefer lower spatial frequencies for B+S-Net than for S-Net, and for 16-class-AlexNet than for 1000-class-AlexNet.

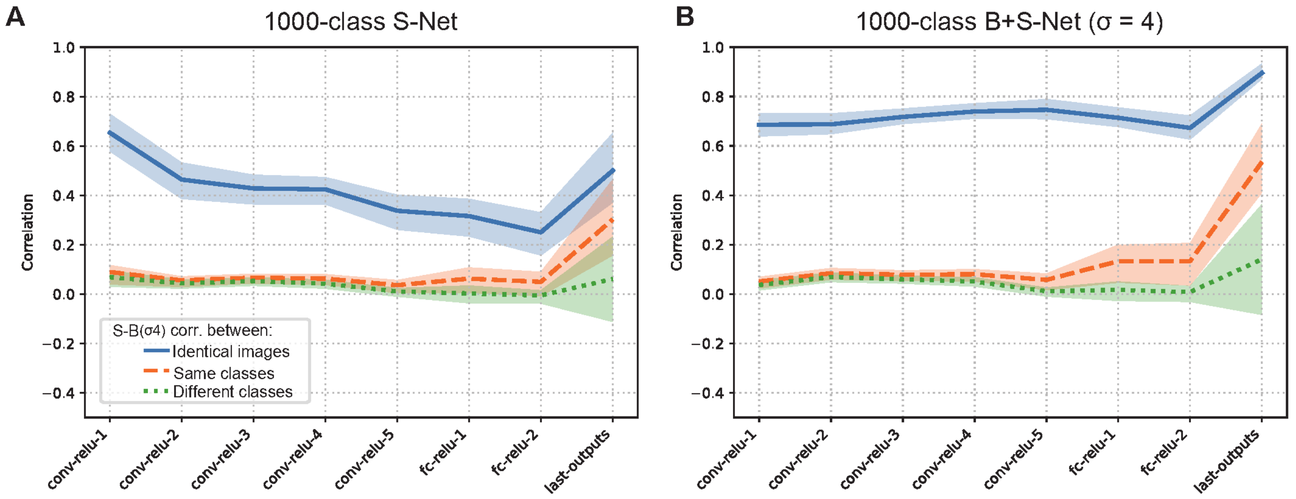


Figure S6: Representational similarity of sharp (unblurred) and blurred image inputs for S-Net (A) and B+S Net (B). The result of 1000-Class AlexNet is shown. The pattern of results is similar to that of 16-class AlexNets in Fig. 5.

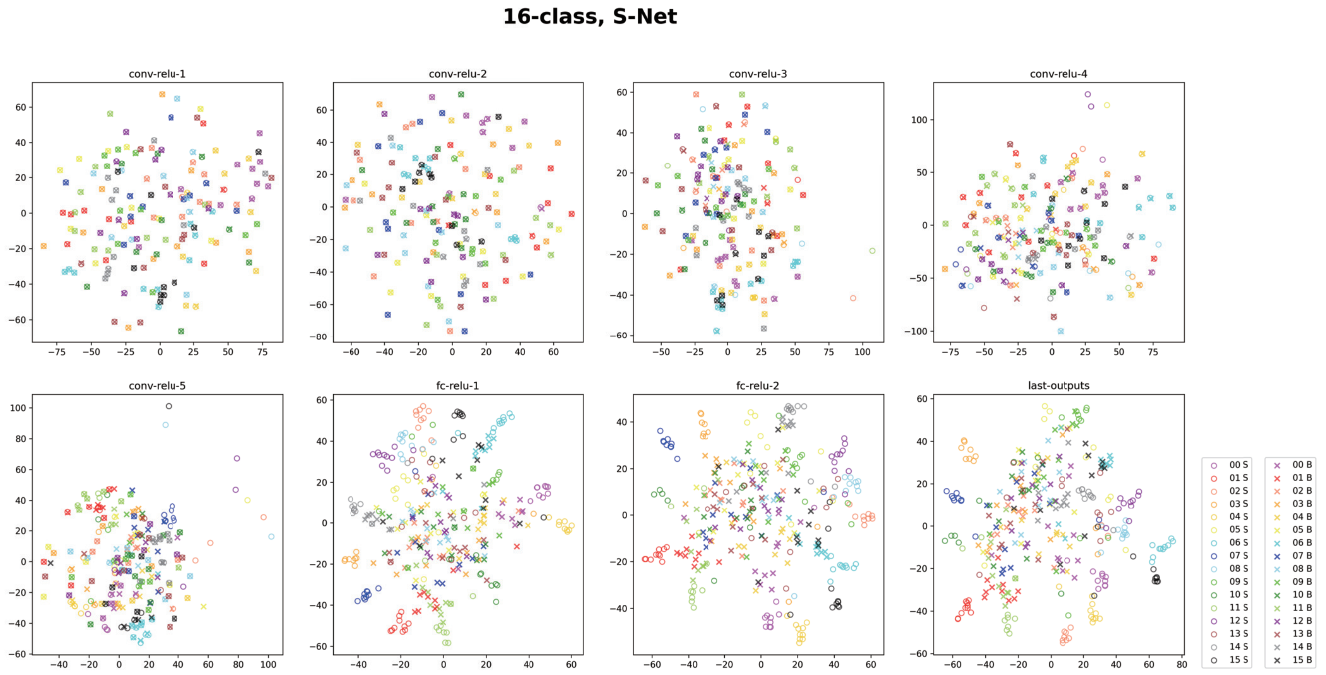


Figure S7: Visualization of internal representation of S-Net (16-class AlexNet) for sharp and blurred images using t-SNE. Each point indicates each of 10 sharp (S) or blurred (B) images of the object class indicated by the two-digit number. 00:airplane, 01:bear, 02:bicycle, 03:bird, 04:boat, 05:bottle, 06:car, 07:cat, 08:chair, 09:clock, 10:dog, 11:elephant, 12:keyboard, 13:knife, 14:oven and 15:truck.

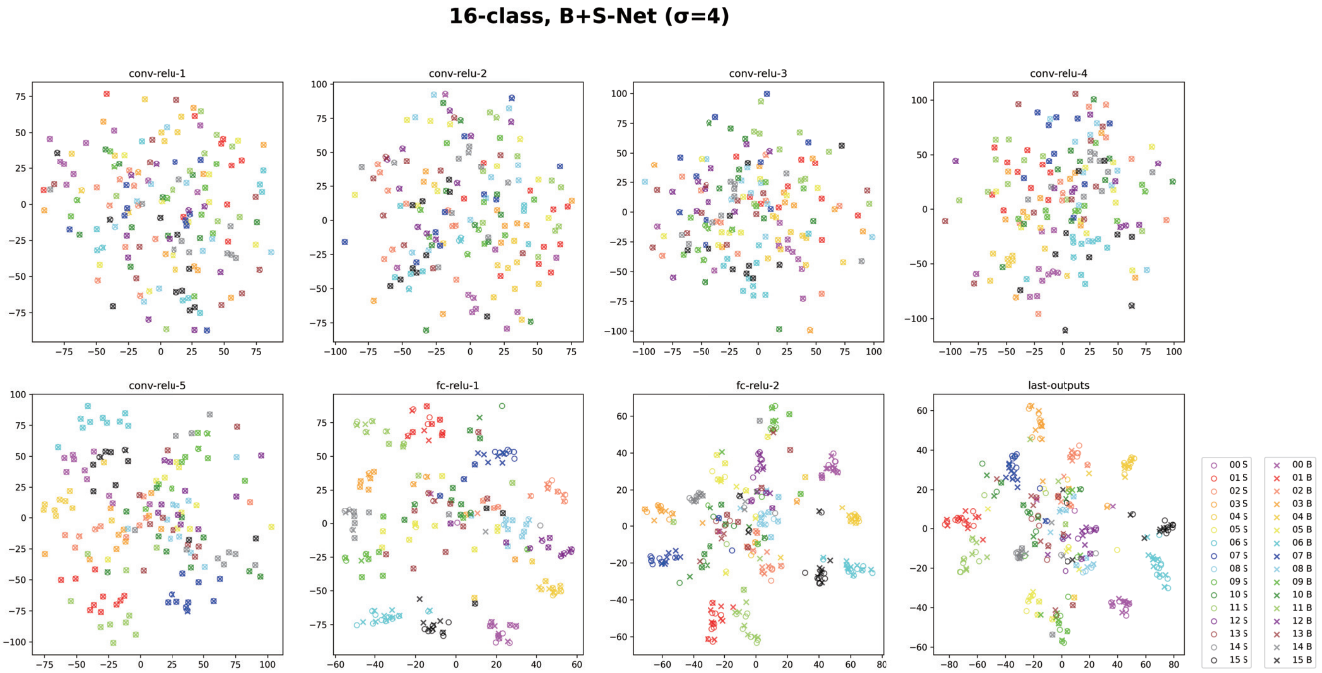


Figure S8: Visualization of internal representation of B+S-Net (16-class AlexNet) for sharp and blurred images by t-SNE. In comparison to S-Net, sharp and blurred images are always close to each other.

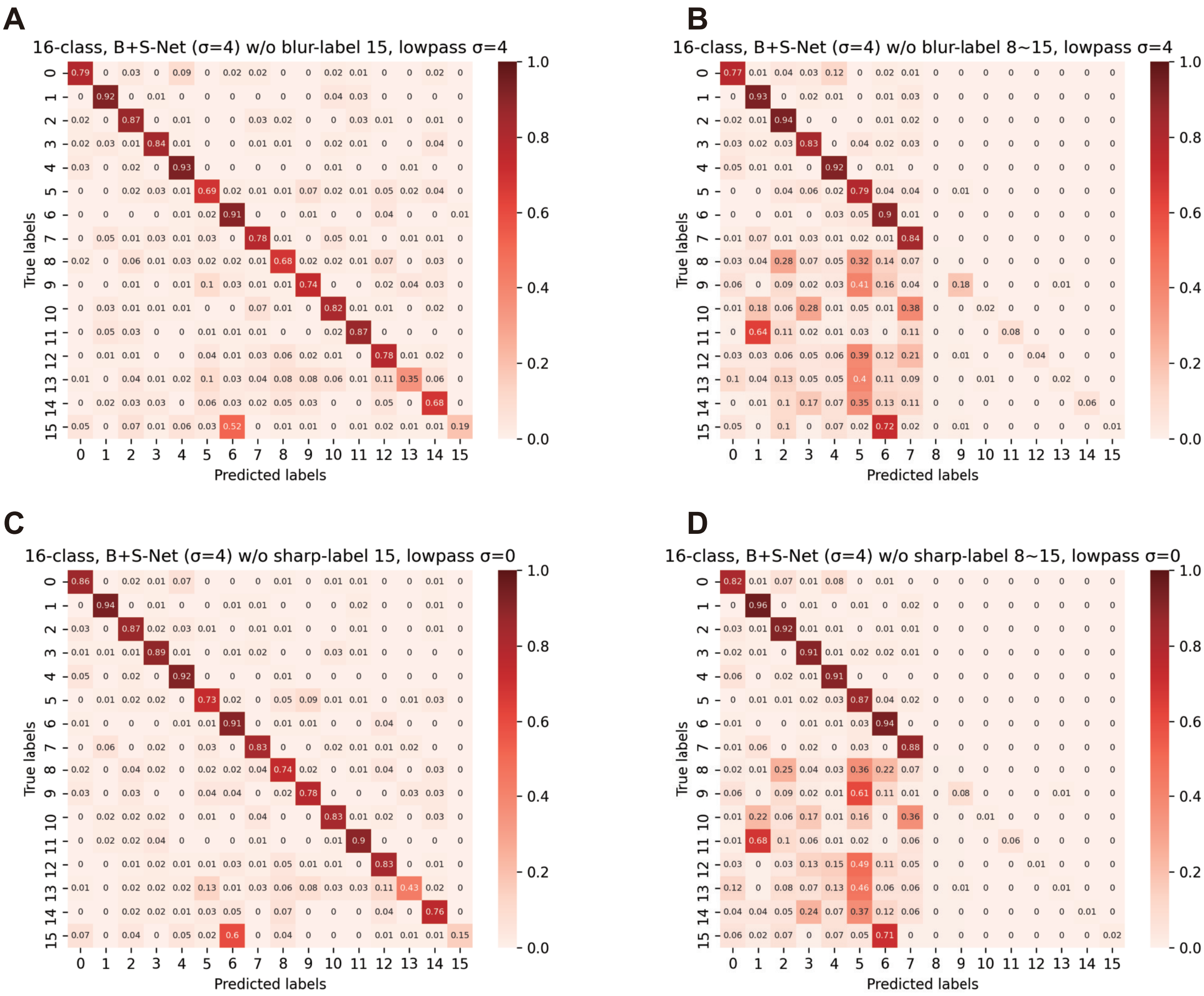


Figure S9: Confusion matrix for zero-shot learning test when (A) one blur label, (B) eight blur labels, (C) one sharp label, or (D) eight sharp labels is/are excluded from the training set. Test set was blurred images for (A) and (B), and sharp images for (C) and (D).

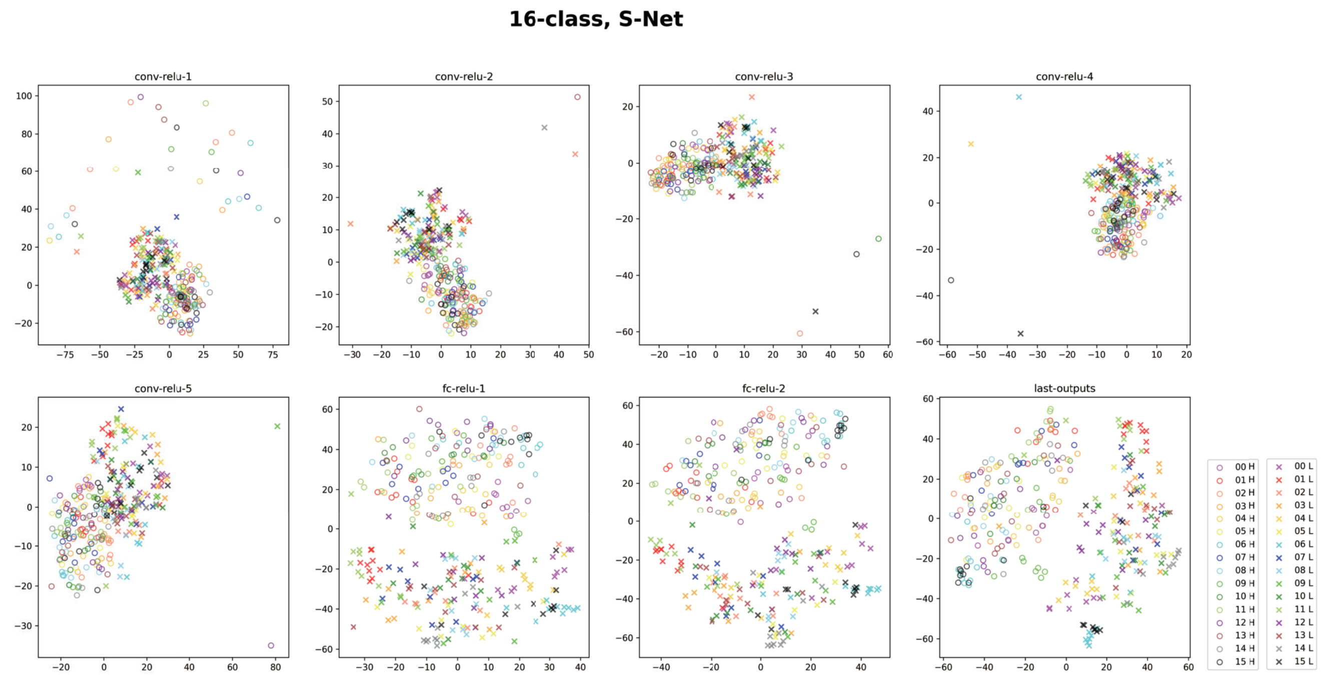


Figure S10: Visualization of internal representation of S-Net (16-class AlexNet) for high-pass and low-pass images by t-SNE. Each point indicates each of 10 high-pass (H) or low-pass (B) images of the object class indicated by the two-digit number.

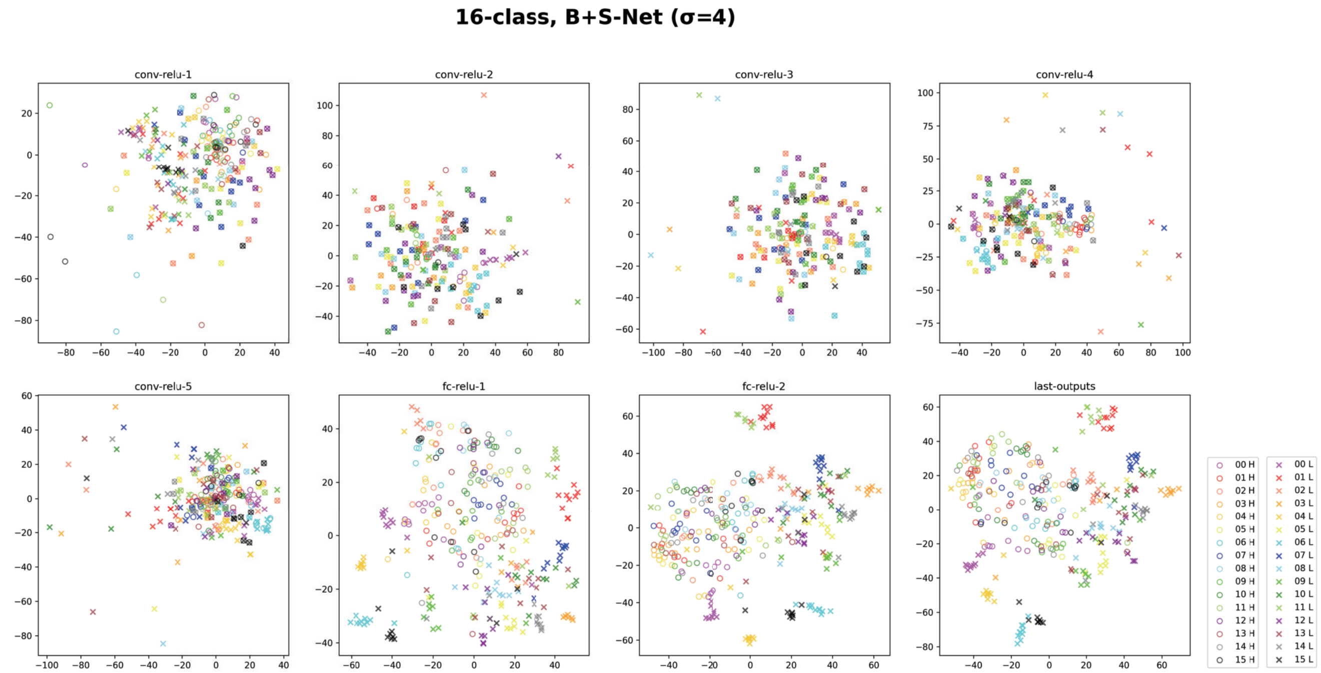


Figure S11: Visualization of the internal representation of B+S-Net (16-class AlexNet) for high-pass and low-pass images by t-SNE.
